# Strategies for meiotic sex chromosome dynamics and telomeric elongation in Marsupials

**DOI:** 10.1371/journal.pgen.1010040

**Published:** 2022-02-07

**Authors:** Laia Marín-Gual, Laura González-Rodelas, Gala Pujol, Covadonga Vara, Marta Martín-Ruiz, Soledad Berríos, Raúl Fernández-Donoso, Andrew Pask, Marilyn B. Renfree, Jesús Page, Paul D. Waters, Aurora Ruiz-Herrera

**Affiliations:** 1 Departament de Biologia Cel·lular, Fisiologia i Immunologia, Universitat Autònoma de Barcelona, Cerdanyola del Vallès, Spain; 2 Genome Integrity and Instability Group, Institut de Biotecnologia i Biomedicina, Universitat Autònoma de Barcelona, Cerdanyola del Vallès, Spain; 3 Departamento de Biología, Facultad de Ciencias, Universidad Autónoma de Madrid, Madrid, Spain; 4 Programa de Genética Humana, Facultad de Medicina, Universidad de Chile, Santiago, Chile; 5 School of BioSciences, The University of Melbourne, Melbourne, Australia; 6 School of Biotechnology and Biomolecular Sciences, Faculty of Science, UNSW Sydney, Australia; The University of North Carolina at Chapel Hill, UNITED STATES

## Abstract

During meiotic prophase I, homologous chromosomes pair, synapse and recombine in a tightly regulated process that ensures the generation of genetically variable haploid gametes. Although the mechanisms underlying meiotic cell division have been well studied in model species, our understanding of the dynamics of meiotic prophase I in non-traditional model mammals remains in its infancy. Here, we reveal key meiotic features in previously uncharacterised marsupial species (the tammar wallaby and the fat-tailed dunnart), plus the fat-tailed mouse opossum, with a focus on sex chromosome pairing strategies, recombination and meiotic telomere homeostasis. We uncovered differences between phylogroups with important functional and evolutionary implications. First, sex chromosomes, which lack a pseudo-autosomal region in marsupials, had species specific pairing and silencing strategies, with implications for sex chromosome evolution. Second, we detected two waves of γH2AX accumulation during prophase I. The first wave was accompanied by low γH2AX levels on autosomes, which correlated with the low recombination rates that distinguish marsupials from eutherian mammals. In the second wave, γH2AX was restricted to sex chromosomes in all three species, which correlated with transcription from the X in tammar wallaby. This suggests non-canonical functions of γH2AX on meiotic sex chromosomes. Finally, we uncover evidence for telomere elongation in primary spermatocytes of the fat-tailed dunnart, a unique strategy within mammals. Our results provide new insights into meiotic progression and telomere homeostasis in marsupials, highlighting the importance of capturing the diversity of meiotic strategies within mammals.

## Author summary

The generation of haploid gametes is a hallmark of sexual reproduction. And this is accomplished by a complex, albeit tightly regulated, reductional cell division called meiosis. Although meiosis has been extensively studied in eutherian mammal model species, our understanding of the mechanisms regulating chromosome synapsis, recombination and segregation during meiosis progression is still incomplete especially in non-eutherian mammals. To fill this gap and capture the diversity of meiotic strategies among mammals, we study previously uncharacterised representative marsupial species, an evolutionary assemblage that last shared a common ancestry more than 80 million years ago. We uncover novel, hence non-canonical, strategies for sex chromosome pairing, DNA repair, recombination and transcription. Most importantly, we reveal the uniqueness of marsupial meiosis, which includes the unprecedented detection of alternative mechanism (ALT) for the paternal control of telomere length during prophase I. Our findings suggest that ALT (previously only associated to cancer cells) could play a role in telomere homeostasis in mammalian germ cells.

## Introduction

A hallmark of sexual reproduction is the generation of haploid gametes with half the chromosome complement of progenitor cells by a complex, albeit tightly regulated, reductional cell division called meiosis. Meiosis generates genetically variable gametes by homologous recombination, which involves faithful chromosome synapsis and DNA exchange between homologous chromosomes during meiotic prophase I. The mechanisms underlying meiotic progression have been extensively studied in model organisms, including yeast, fruit flies, nematodes, mice and, more recently, zebrafish [1,2]. This has revealed canonical features that are conserved across large evolutionary time scales, including fundamental events such as the formation of double strand breaks (DSBs—essential for meiotic recombination), homologous chromosome pairing and synapsis, and the formation of the telomeric bouquet. However, important differences between taxa have been noted, highlighting that our understanding of mammal meiotic prophase I is still incomplete, especially in non-traditional model species [3].

Mammals (represented by monotremes, marsupials and eutherians) last shared a common ancestor approximately 185 million years ago (Mya) [4] and are characterised by distinctive genome plasticity [5,6]. Despite genome reshuffling, canonical features of the meiotic programme are well conserved in eutherian mammals (i.e., human, non-human primates, rodents and bovids) [3,7–12]. In contrast, detailed immunofluorescence studies on chromosome pairing during prophase I in marsupials are scarce and restricted to a handful of American species [13,14]. Due to their distant relationship with eutherian mammals, and that Australian and American species shared a common ancestor 80 Mya [15], marsupials offer a unique opportunity to explore previously uncharacterised meiotic features. This includes unique sex chromosome pairing strategies, recombination and meiotic telomeric homeostasis.

One exceptional property of marsupial sex chromosomes is that, unlike eutherian sex chromosomes, the X and Y chromosomes do not share a homologous region within which recombination occurs (i.e., pseudo-autosomal region or PAR) [16]. As a consequence, sex chromosomes associate during prophase I via a marsupial specific structure called the dense plate (DP) [13,17,18], which is rich in synaptonemal complex proteins and ensures faithful segregation in the absence of synapsis and recombination.

**Fig 1:**
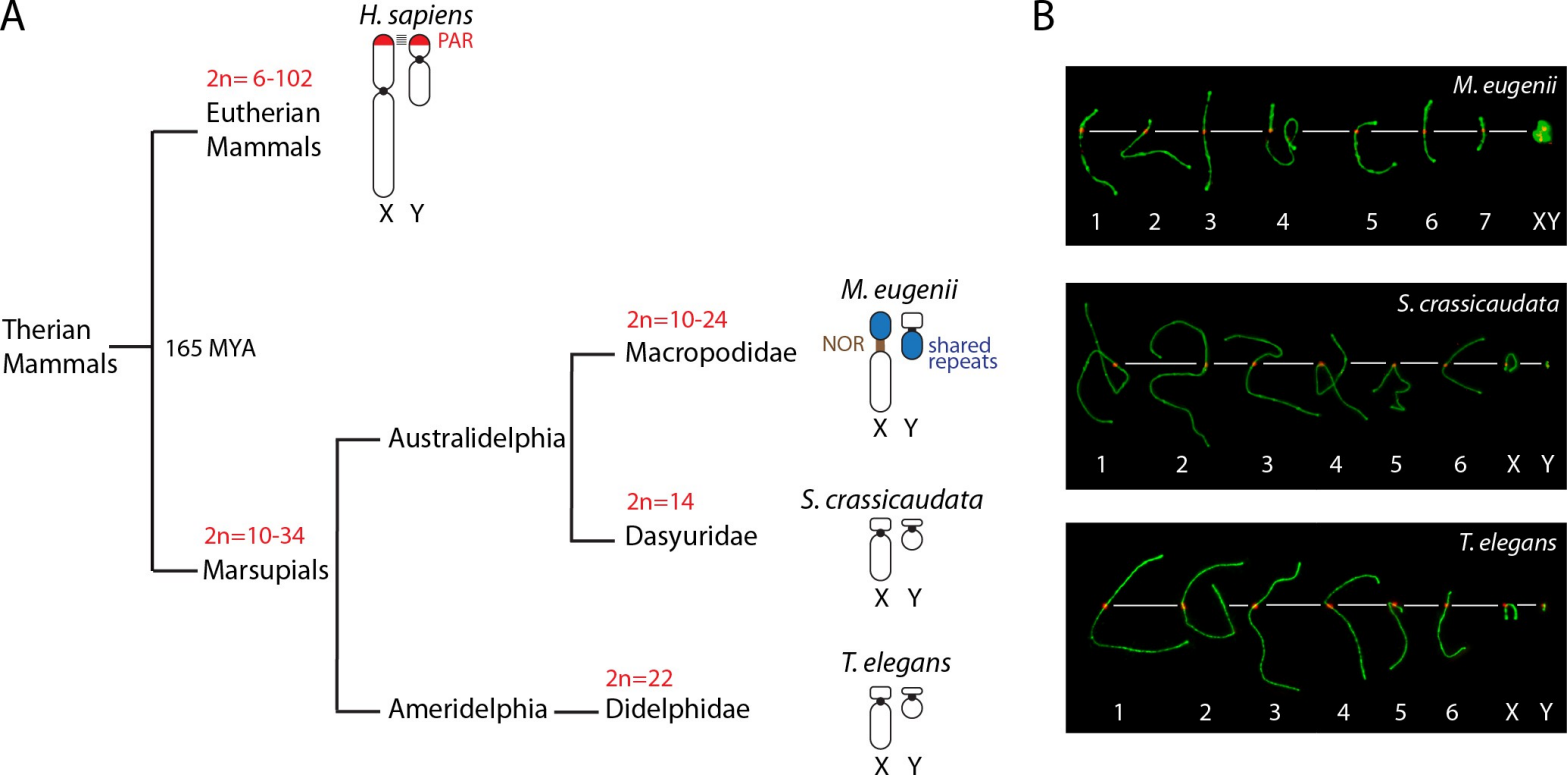
Phylogeny of the marsupial species included in the study. (A) Phylogenetic relationships of the three marsupial species included in the study, with representation of sex chromosome structure for each species. Human sex chromosomes are included for comparison. Variation in diploid numbers is indicated for each phylogenetic branch. All marsupials lack a pseudo-autosomal region (PAR). The tammar (*M*. *eugenii*) X chromosome is large compared to the fat-tailed dunnart (*S*. *crassicaudata*) and the fat-tailed mouse opossum (*T*. *elegans*). Moreover, the tammar X chromosome bears NOR sequences in the centromeric region and the p-arm contains a region of shared DNA repeats with the q-arm of the Y chromosome [31]. (B) Meiotic karyotypes of the species included in the study: *M*. *eugenii*, *S*. *crassicaudata* and *T*. *elegans*. Karyotypes correspond to primary spermatocytes at pachytene labelled with antibodies against SYCP3 (green) and centromeres (red). The tammar sex chromosomes form a highly condensed dense plate at pachytene.

A salient feature of sex chromosomes is their transcriptional silencing during prophase I–a phenomenon called meiotic sex chromosome inactivation (MSCI) [19,20]. MSCI is a specialization of the MSUC (meiotic silencing of unsynapsed chromatin) process and it is characterised by accumulation of chromatin modifications in response to asynapsed chromatin during prophase I, including the phosphorylation of histone H2AX on serine 139 (γH2AX) [21–24]. MSCI is restricted to the heterogametic sex in species with heteromorphic sex chromosomes, and is a conserved epigenetic silencing programme in therian mammals [25–28]. It is a meiotic checkpoint that detects the presence of partial or completely unsynapsed homologous chromosomes, which results in the inactivation of ‘pachytene-lethal’ genes on the Y chromosome [29,30]. But, not all marsupials have the same sex chromosome structure [18], suggesting that there could be requirement for different meiotic pairing and silencing strategies. The tammar wallaby (and other macropods) are characterised by the X chromosome bearing a nucleolus organising region (NOR) near the centromere, along with a recently acquired satellite repeat region on the p-arm that is shared with the q-arm of the Y chromosome (Fig 1) [18,31]. This is considered a derived state [31,32] as dasyurid marsupials (e.g., Tasmanian devil, quolls and dunnarts) have a conserved X chromosome structure that is shared with American marsupials [15].

Moreover, dasyurids are characterised by an extreme telomere length dimorphism between homologous chromosomes, which evolved before the dasyurid radiation at least 50 Mya [33,34]. Initial observations in male dasyurids showing that Y chromosomes had long telomeres and X chromosomes had short telomeres, suggested that telomere length dimorphism was due to a parental-of-origin effect, with long telomeres inherited from the paternal germline and short telomeres from the maternal germline [33]. This begs for description of a novel strategy of telomere length homeostasis in the parental germline. When and where in the male germline telomeres are elongated needs experimental validation.

Here we provide new insights into key features of meiotic prophase I progression in previously uncharacterised Australian marsupial linages, including an American taxon for comparison (Fig 1). We examined the tammar wallaby (*Macropus eugenii*) a representative of Macropodidae, the fat-tailed dunnart (*Sminthopsis crassicaudata*) a representative of Dasyuridae, and the fat-tailed mouse opossum (*Thylamys elegans*) a representative of Didelphidae that is endemic to the Americas. We showed sex chromosomes pairing configurations during prophase I that are distinct between species, and described that there were lower levels of γH2AX on autosomes than sex chromosomes, most probably due to low rates of DSB formation. Importantly, we detected that telomeres were actively transcribed and elongated during prophase I in the fat-tailed dunnart, resetting all paternally inherited telomeres so that they are long in sperm.

## Results

### Sex chromosome meiotic pairing strategies in marsupials

The chromosome complement of tammar wallaby is 2n = 16, whereas both fat-tailed mouse opossum and fat-tailed dunnart are characterised by 2n = 14 (Fig 1). Differences in diploid numbers are mainly due to lineage-specific chromosome rearrangements in macropods [35], highlighting the derivative state of this clade of Australian marsupials.

**Fig 2:**
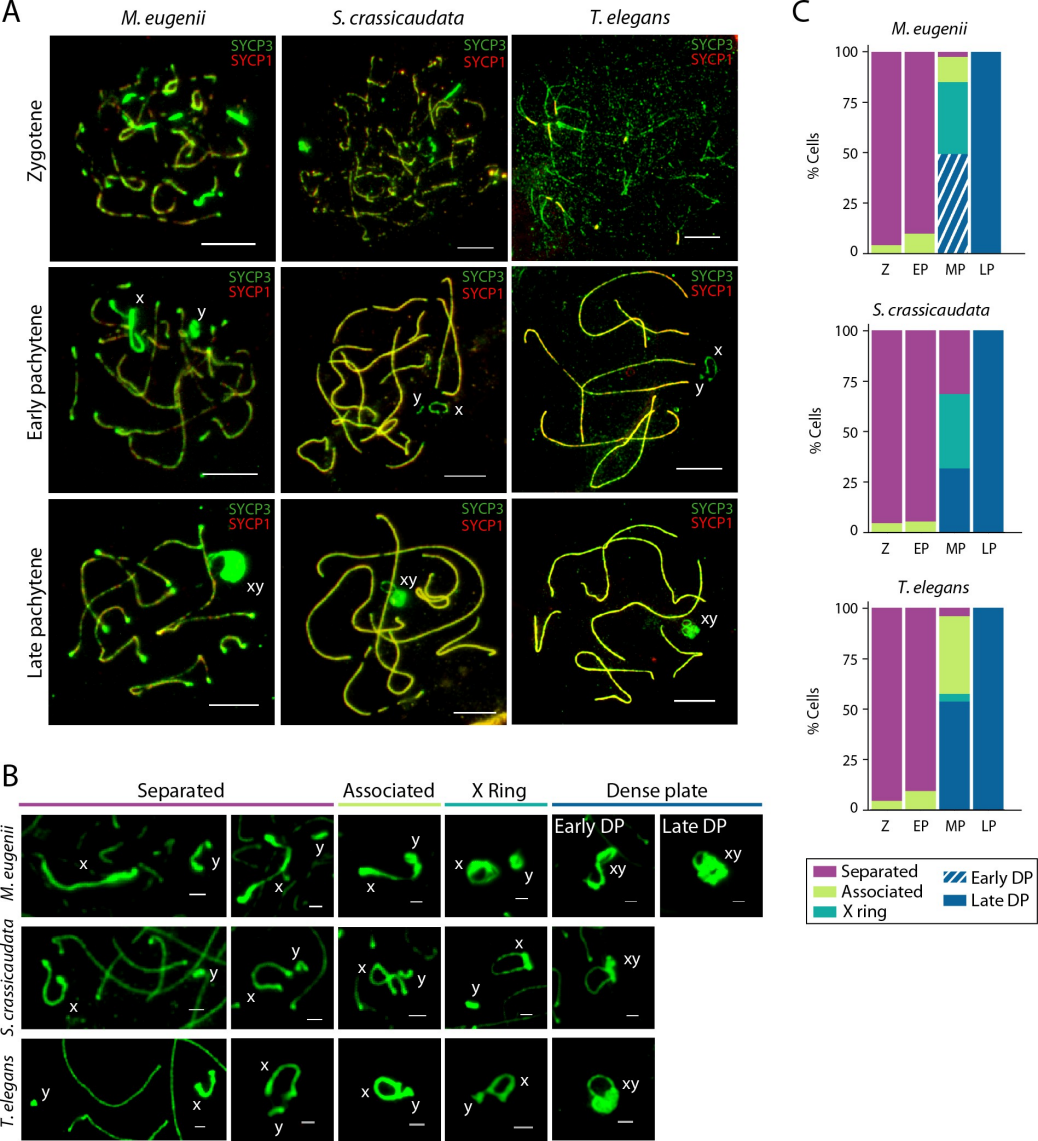
Pairing dynamics during prophase I. (A) Spermatocyte spreads in prophase-I labelled with antibodies against SYCP3 (green) and SYCP1 (red) for the tammar wallaby (*M*. *eugenii*), the fat-tailed dunnart (*S*. *crassicaudata*), and the fat-tailed mouse opossum (*T*. *elegans*). Scale bar = 10μm. (B) Sex chromosomes pairing configurations during prophase I (separated, associated, X ring or DP) for tammar wallaby, fat-tailed dunnart, and fat-tailed mouse opossum. In tammar wallaby the DP can adopt two different configurations: the early DP has an open configuration, whereas the late DP is compacted. Scale bar = 2μm. (C) Percentage of cells with different sex chromosomes configurations for primary spermatocytes from tammar wallaby (N = 41 cells in zygotene, N = 19 cells in early pachytene, N = 49 cells in mid pachytene and N = 81 cells in late pachytene), fat-tailed dunnart (N = 22 cells in zygotene, N = 19 cells in early pachytene, N = 19 cells in mid pachytene and N = 28 cells in late pachytene), and fat-tailed mouse opossum (N = 23 cells in zygotene, N = 22 cells in early pachytene, N = 161 cells in mid pachytene and N = 168 cells in late pachytene). Cell type legend: Z, zygotene; EP, early pachytene; MP, mid pachytene; LP, late pachytene.

Anti-SYCP3 antibody labelled axial elements of the synaptonemal complex were used to classify spermatocytes into the different prophase I stages, following previous observations in marsupials [13,14] (Fig 2). At leptotene, short filaments of SYCP3 were observed in the three species, representing the forming axial elements (S1 Fig). Chromosome ends appear clustered in a bouquet configuration (S1A Fig). Axial elements become larger at zygotene, when synapsis between homologous chromosomes takes place, as revealed by SYCP3 and SYCP1 labelling (Fig 2A). The distinction between early and late zygotene was based on the relatively length of discontinuous axial structures. At pachytene, autosomes have completed synapsis. Spermatocytes at pachytene were divided into three sub-stages (early, mid and late) based on the previously described structure and behaviour of sex chromosomes [13]. Briefly, at early pachytene sex chromosomes were separated with thick axial element labelling (SYCP3 signal). At mid pachytene sex chromosomes became associated, had thinner axial elements than autosomes and the DP was beginning to form. Late pachytene was distinguished by the presence of a fully developed DP.

The general trend in all three marsupials was for the X and Y to associate after autosomes had paired (Figs 2 and S1B–S1D). During mid pachytene the sex chromosomes approach each other to form the DP, adopting four possible configurations that were classified as the following: (i) separated–sex chromosomes not in contact, (ii) associated–sex chromosomes in contact but DP not formed, (iii) ‘X ring’–the X chromosome forms a ring while approaching the Y, along with thickening of their axes without forming the DP, and (iv) DP–sex chromosomes come together and the DP forms (Fig 2B). In tammar, the DP adopted two further configurations: an early DP with an open configuration, and a late DP with a more compacted structure (Fig 2B).

Although these four sex chromosomes pairing configurations were present in all three marsupials, differences in structure were apparent in early pachytene. Separated X and Y chromosomes in tammar wallaby showed thicker axial axes (SYCP3 labelling) than the fat-tailed mouse opossum and dunnart (Fig 2A and 2B). As sex chromosomes approach each other in early pachytene, the tammar X chromosome remain in a ‘stretched’ configuration. This contrasted the fat-tailed mouse opossum and dunnart, in which telomeres of the X chromosome were close to each other (Fig 2B). Whereas the fat-tailed mouse opossum and dunnart formed a neat and clear ring, the tammar X associated in a large, intensely stained irregular structure. This was not resolved until formation of the DP, which was also different in configuration when compared to the non macropod species (Fig 2B). These results mirror early electron microscopy observations [18], and are consistent with the pattern detected in *T*. *elegans* and other American species [14].

Moreover, we found differences in the proportions of pairing configurations as prophase I progressed, particularly during mid pachytene (Fig 2C). The fat-tailed mouse opossum (the American marsupial representative) presented very few cells with X chromosomes forming rings in mid pachytene (3.7% cells). This contrasted both Australian representatives (tammar wallaby and fat-tailed dunnart), where 34.7% and 36.8% of cells, respectively, presented X rings. Collectively, these results suggest differences in the timing of sex chromosomes association and DP formation.

### Sex chromosome transcription in the tammar wallaby

**Fig 3:**
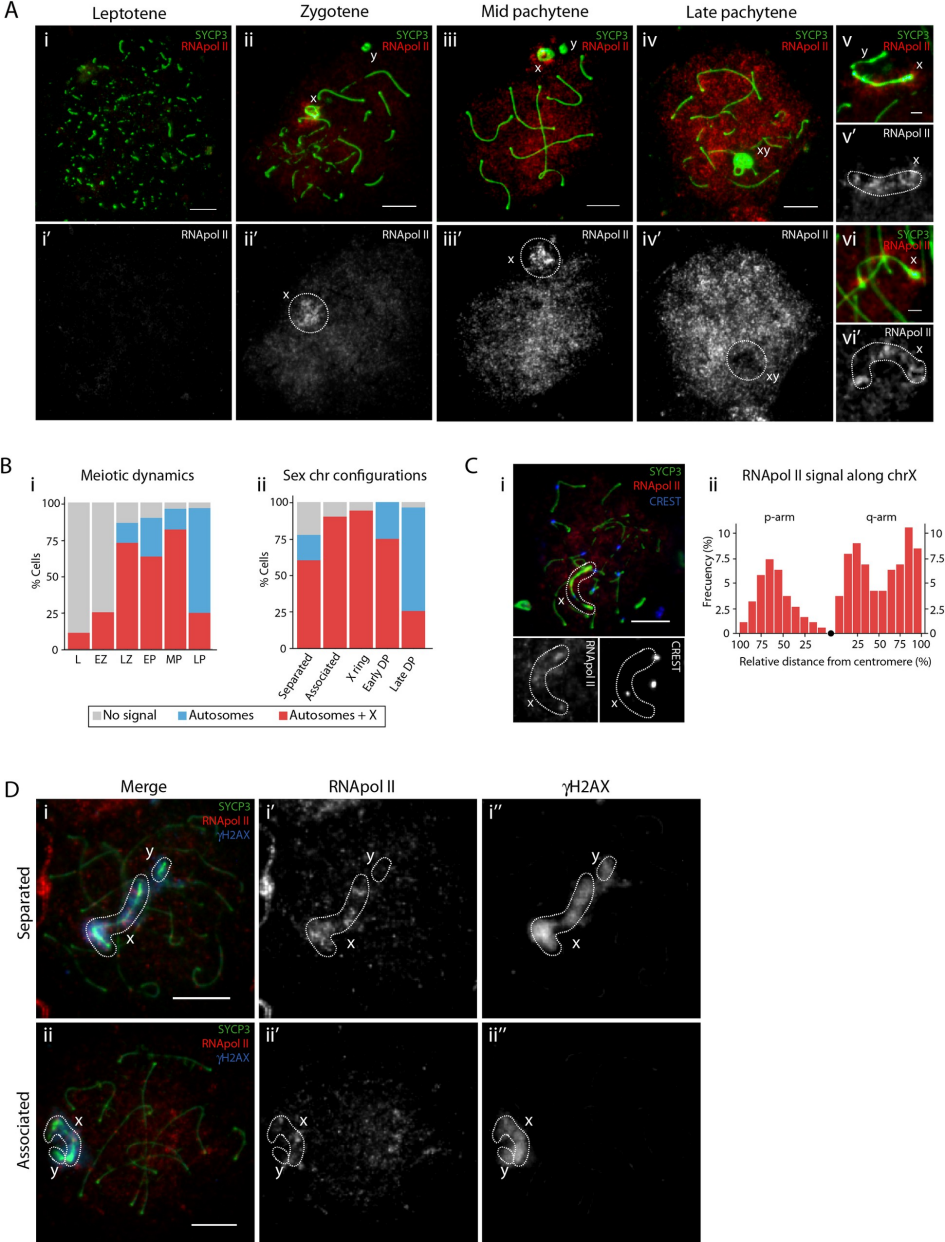
Transcription dynamics during prophase I in the tammar wallaby. (A) Representative images of tammar wallaby (*M*. *eugenii*) spermatocyte spreads in prophase-I labelled with antibodies against SYCP3 (green) and RNA pol II (red). Scale bar = 10μm. (v-vi) Active transcription of the X chromosome with an open configuration. Scale bar = 2μm. White dashed circles: RNA pol II signal on the X chromosome. (B) (i) Percentage of cells with different transcription patterns for spermatocytes in leptotene (N = 9 cells), early zygotene (N = 24 cells), late zygotene (N = 29 cells), early pachytene (N = 19 cells), mid pachytene (N = 49 cells) and late pachytene (N = 81 cells). (ii) Percentage of cells with different transcription patterns for spermatocytes with different sex chromosome configurations: separated (N = 58), associated (N = 10 cells), X ring (N = 17 cells), early DP (N = 24 cells) and late DP (N = 81 cells). (C) Transcription along the X chromosome. (i) Spermatocyte spread labelled with antibodies against SYCP3 (green), RNA pol II (red) and centromeres (blue). White dashed circles: X chromosome. Scale bar = 10μm. (ii) Distribution of the RNApol II signal along X chromosomal arms. The X axis represents the positions on the chromosomal axes from the centromeric end (black dot) to p-arm and q-arm telomeres. The Y axis indicates the frequency of RNA pol II signal detected for each 10% interval of chromosomal length (N = 30 cells). Black dot: centromere. (D) Co-localization of RNA pol II and γH2AX. Spermatocyte spreads labelled with antibodies against SYCP3 (green), RNA pol II (red) and γH2AX (blue). White dashed circles: sex chromosomes. Scale bar = 10μm.

**Fig 4:**
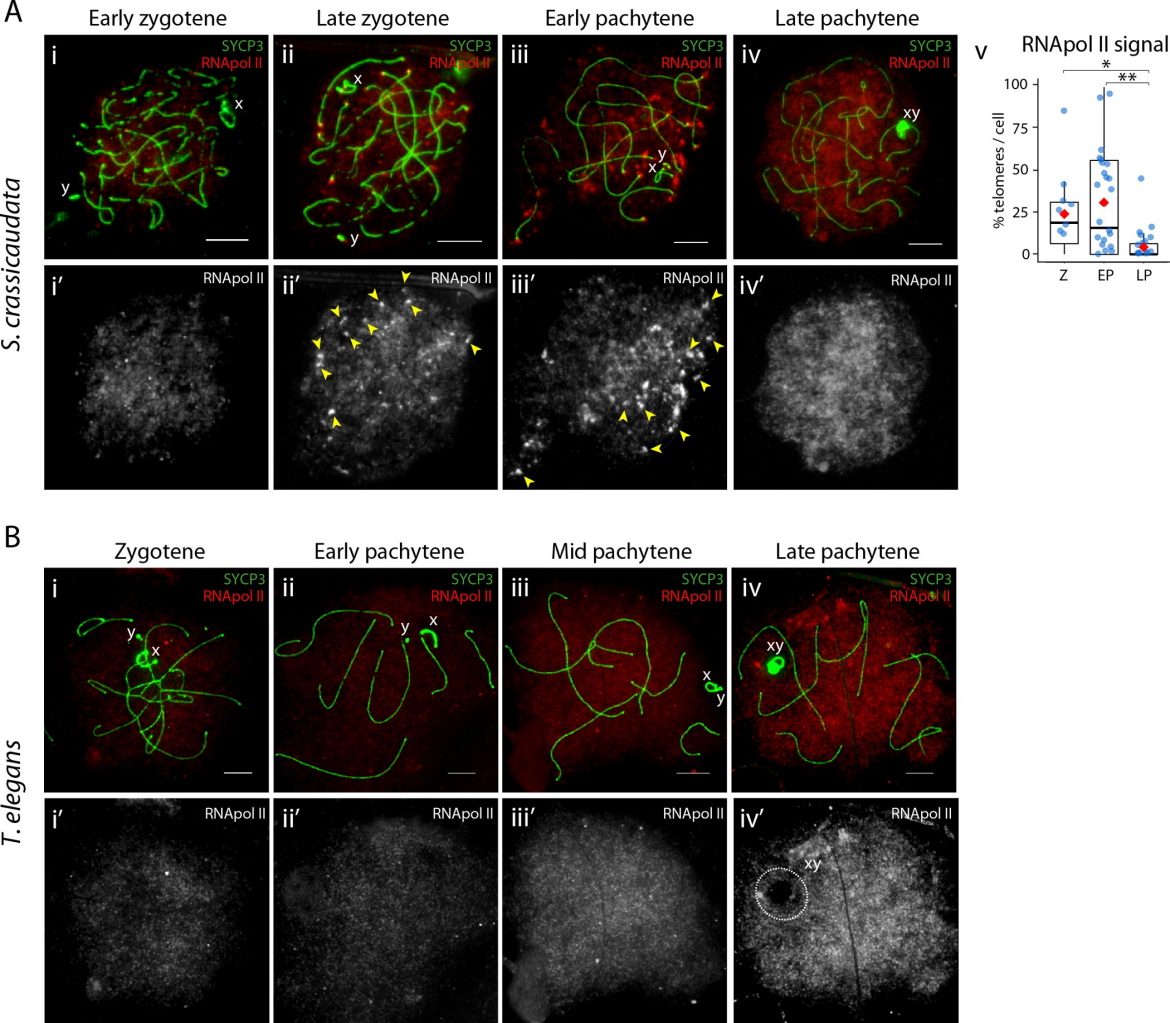
Transcription dynamics during prophase I in the fat-tailed dunnart and the fat-tailed mouse opossum. (A) Representative images of fat-tailed dunnart (*S*. *crassicaudata*) spermatocyte spreads in prophase-I (i-iv) labelled with antibodies against SYCP3 (green) and RNA pol II (red). Scale bar = 10μm. Yellow arrowheads: RNA pol II foci overlapping telomeres. (v) Boxplots showing the percentage of telomeres with RNA pol II signal per cell in spermatocytes in zygotene (N = 11 cells), early pachytene (N = 28 cells) and late pachytene (N = 28 cells). Red diamonds indicate mean value. Wilcoxon pairwise test (*p<0.05, **p<0.01). (B) Representative images of fat-tailed mouse opossum (*T*. *elegans*) spermatocyte spreads in prophase-I labelled with antibodies against SYCP3 (green) and RNA pol II (red). Scale bar = 10μm. White dashed circles: RNA pol II signal in the DP. Cell type legend: Z, zygotene; EP, early pachytene; LP, late pachytene.

Remarkably, in tammar wallaby phosphorylated RNA pol II (the active form of RNA pol II) was observed on the X chromosome (but not the Y) from late zygotene through to mid pachytene (Fig 3). This active transcription of the X chromosome was observed on all four sex chromosome configurations (separated, associated, X ring and early DP) (Fig 3A). RNA pol II was most predominant in late zygotene (72% cells analysed), and surprisingly, during early (63% cells analysed) and mid pachytene (81% cells analysed) (Fig 3B). This contrasts the fait-tailed dunnart and fait-tailed mouse opossum Xs, which were subject to MSCI. In these two species, sex chromosomes remained silent through all of prophase I, with no RNA pol II signal observed on the sex body from mid pachytene onwards (Fig 4).

Tammar wallaby X chromosome transcription was significantly associated with open sex chromosome configurations (*χ2* test, p< 0.05) (Fig 3B and S1 Table). At fine scale, we detected three regions on the X chromosome with concentrated RNA pol II signal (Fig 3C). Only when sex chromosomes formed a condensed DP (i.e. late DP) in late pachytene was the sex body devoid of an RNA pol II signal, although 25% of spermatocytes still showed a signal at this stage (Fig 3B). This change in chromosome conformation was concomitant with MSCI through the remainder of prophase I (Fig 3A).

Our results suggest that differences in the timing of meiotic pairing dynamics and sex chromosome configurations, especially in tammar wallaby, can be accounted for differences in chromosome architecture and transcription. The sex chromosomes in dasyurids (i.e., fat-tailed dunnart) and didelphids (i.e., fat-tailed mouse opossum) lack a PAR and are small [13,36], whereas in tammar wallaby the X is larger, bearing a NOR and XY shared repetitive sequence, which are unique to macropods (Fig 1). As the detection of RNA pol II signal was associated with ‘stretched’ X configurations in tammar, it is tempting to speculate that possible open chromosome configurations are necessary for expression during a specific window in prophase I.

### Localisation of γH2AX on sex chromosomes and transcription are not mutually exclusive in tammar wallaby

**Fig 5:**
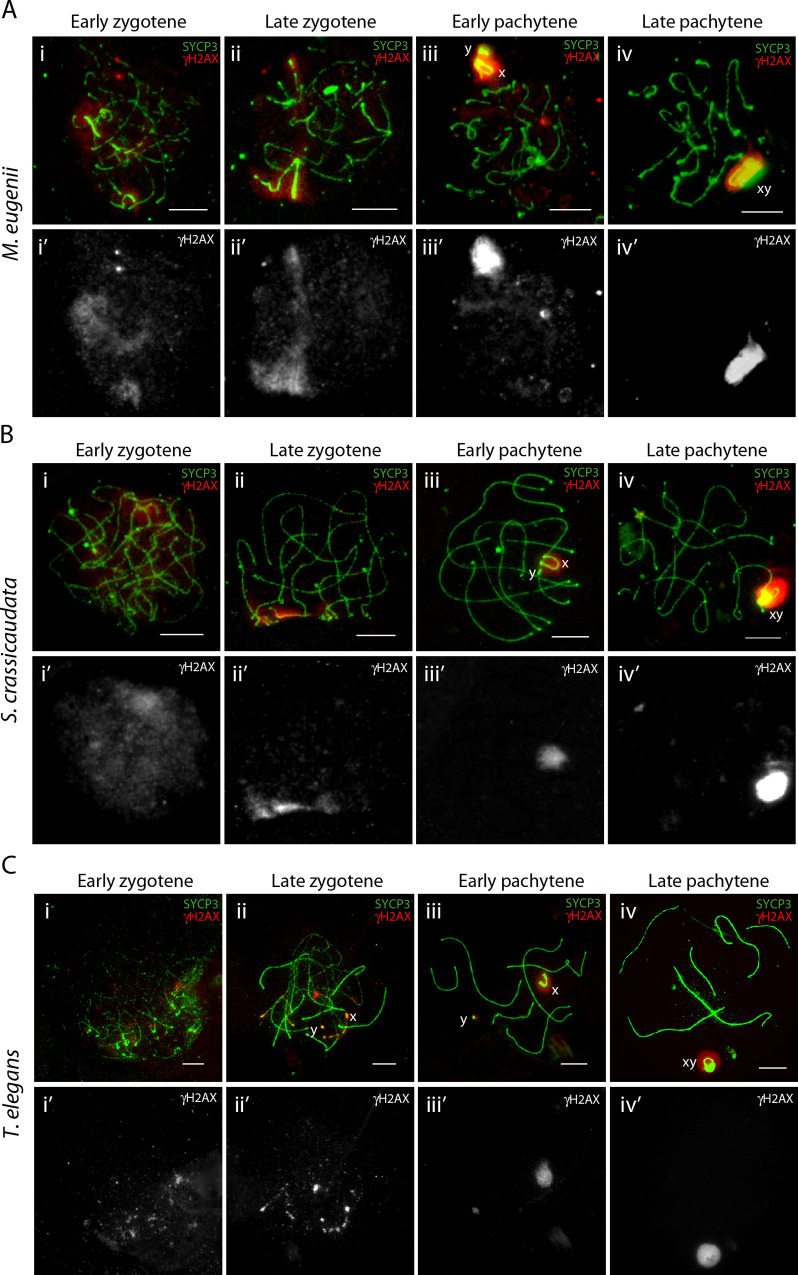
γH2AX progression during prophase-I in marsupials. Spermatocyte spreads labelled with antibodies against SYCP3 (green) and γH2AX (red) for (A) tammar wallaby (*M*. *eugenii*), (B) fat-tailed dunnart (*S*. *crassicaudata*) and (C) fat-tailed mouse opossum (*T*. *elegans*). Scale bar = 10μm.

We then studied the dynamics of γH2AX on sex chromosomes, as it is known to be associated with MSCI in eutherians [25]. During leptotene and zygotene we detected scarce and faint γH2AX signals in the whole nucleus in all three marsupial species (Fig 5). As sex chromosomes approached each other during late zygotene and pachytene, the γH2AX signal was restricted to both the X and Y chromosomes, forming discrete chromatin domains, even if the chromosomes were located at opposite poles of the cell (Fig 5). Gamma H2AX displayed a stronger signal in late pachytene, concomitant with the formation of the DP in all three species (Fig 5), mirroring previous observations [26,28].

Crucially, in tammar wallaby the γH2AX signal collocated with the phosphorylated RNA pol II signal on the X chromosome during zygotene (mainly late zygotene) and early/mid pachytene, before depletion of RNA pol II at late pachytene (Fig 3D) and formation of a closed chromatin domain (Fig 5). Therefore, γH2AX signal was detected on sex chromosomes even though they were physically separated and the X chromosome transcriptionally active (Figs 3 and 5). It was not until formation of the DP in late pachytene, when sex chromosomes were devoid of RNA pol II signal and the γH2AX signal was more intense. This suggests that RNA (either coding or non-coding/repetitive regions) transcribed from the X chromosome (p- and q-arm) escape silencing in tammar until DP formation and MSCI initiation in late pachytene.

### Low levels of γH2AX on autosomes are associated to low rates of meiotic DSB formation in marsupials

Previous modelling and experimental approaches have shown that marsupials are characterised by lower recombination rates than eutherian linages such as Carnivora, Perissodactyla and Cetartiodactyla [3,12]. Therefore, we investigated the cellular mechanisms behind this pattern in our marsupial representatives.

**Fig 6:**
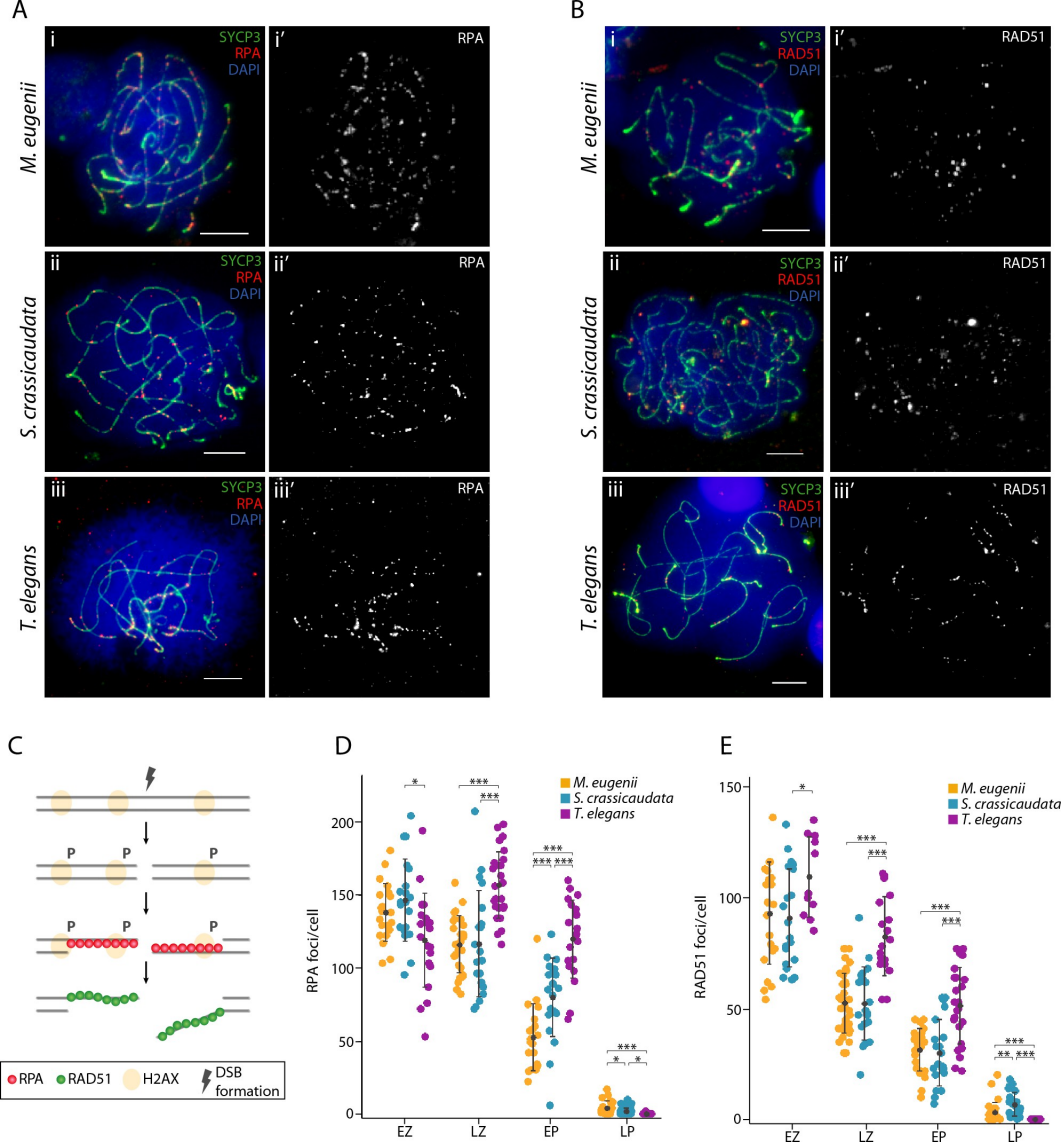
RPA and RAD51 dynamics during prophase I in marsupials. (A) Late zygotene spermatocyte spreads labelled with antibodies against SYCP3 (green) and RPA (red) for all three marsupial species, labelling the DNA with DAPI (blue). Scale bar = 10μm. (B) Late zygotene spermatocyte spread labelled with antibodies against SYCP3 (green) and RAD51 (red) for all three marsupial species, labelling the DNA with DAPI (blue). Scale bar = 10μm. (C) Schematic representation of the proteins involved in the formation and repair of meiotic DSBs. (D) Plot representing the number of RPA foci per cell detected at early zygotene (N = 21 cells in tammar, N = 20 cells in fat-tailed dunnart, N = 20 cells in fat-tailed mouse opossum), late zygotene (N = 26 cells in tammar, N = 20 cells in fat-tailed dunnart, N = 27 cells in fat-tailed mouse opossum), early pachytene (N = 20 cells in tammar, N = 21 cells in fat-tailed dunnart, N = 20 cells in fat-tailed opossum) and late pachytene (N = 21 cells in tammar, N = 30 cells in fat-tailed dunnart, N = 15 cells in fat-tailed opossum). Wilcoxon pairwise test (*p<0.05, **p<0.01, ***p<0.001). (E) Plot representing the number of RAD51 foci per cell detected at early zygotene (N = 22 cells in tammar, N = 20 cells in fat-tailed dunnart, N = 12 cells in fat-tailed mouse opossum), late zygotene (N = 39 cells in tammar, N = 20 cells in fat-tailed dunnart, N = 18 cells in fat-tailed mouse opossum), early pachytene (N = 25 cells in tammar, N = 21 cells in fat-tailed dunnart, N = 24 cells in fat-tailed mouse opossum) and late pachytene (N = 33 cells in tammar, N = 23 cells in fat-tailed dunnart, N = 11 cells in fat-tailed opossum). Wilcoxon pairwise test (*p<0.05, **p<0.01, ***p<0.001). Legend: EZ: early zygotene, LZ: late zygotene, EP: early pachytene, LP: late pachytene.

We analysed the dynamics of DSB formation by immunodetection of the recombination proteins RPA (replication protein A) and RAD51 (radiation sensitive 51) on spermatocyte spreads (Figs 6, S2, S3 and S4). RPA binds to the 3’ strand following DSB formation and consequently accumulates at these sites [37,38] (Fig 6). Then, RPA is displaced by RAD51 and/or DMC1 [39,40], which form nucleoprotein filaments that catalyse strand invasion. Therefore, the number of RPA and RAD51 sites in early prophase is a proxy for the number of DSBs.

Concordant with early genetic linkage maps showing low levels of recombination in marsupials [41], we detected low numbers of RPA and RAD51 foci per cell in all three species (Figs 6, S2, S3 and S4). For RPA, there was an equivalent mean number of foci per cell in both Australian species in early (137.9±28.2 in tammar wallaby and 146.3±19.68 in fat-tailed dunnart) (Mann-Whitney test, p = 0.35) and late (116.04±19.71 in tammar wallaby and 116.5±36.23 in fat-tailed dunnart) (Mann-Whitney test, p = 0.63) zygotene (Fig 6A and 6D). In contrast, RPA loading was lower in the fat-tailed mouse opossum (American representative), with fewer RPA foci per cell in early zygotene (119.05±32.16) (Mann-Whitney test, p<0.05). However, the fat-tailed mouse opossum had a higher mean number of RPA foci by late zygotene (156.7±22.64) (Mann-Whitney test, p<0.001) compared to both Australian marsupials.

RPA foci number decreased as they were replaced by RAD51 during DSB repair, reaching a minimum in late pachytene (4.29±4.73 in tammar wallaby, 1.7±2.55 in fat-tailed dunnart and 0.13±0.52 in fat-tailed mouse opossum). The replacement dynamic was different between the Australian and American representatives, with a higher number of RPA foci per cell in early pachytene in the fat-tailed mouse opossum (119.8±26.68) compared to fat-tailed dunnart (79.9±26.95) and tammar wallaby (52.5±22.79) (Mann-Whitney test, p<0.001) (Fig 6D).

A similar trend was observed for the mean number of RAD51 foci per cell in Australian marsupials, with lower mean values compared to the fat-tailed mouse opossum in early zygotene (93.00±23.03 in tammar wallaby, 90.85±22.11 in fat-tailed dunnart and 112.42±20.32 in fat-tailed mouse opossum) (Mann-Whitney test, p<0.05), late zygotene (52.46±13.37 in tammar wallaby, 52.25±16.58 in fat-tailed dunnart and 82.56±17.83 in fat-tailed mouse opossum) (Mann-Whitney test, p<0.001) and early pachytene (31.36±9.62 in tammar wallaby, 30.05±14.98 in fat-tailed dunnart and 51.33±17.15 in the fat-tailed mouse opossum) (Mann-Whitney test, p<0.001) (Fig 6B and 6E). Differences between the two Australian marsupials were only detected in late pachytene (6.91±5.26 in fat-tailed dunnart and 3.21±4.40 in tammar wallaby) (Mann-Whitney test, p<0.05). However, both had higher mean values than the fat-tailed mouse opossum (Mann-Whitney test, p<0.001) (Fig 6E). RAD51 foci in prophase I were concentrated on the sex chromosomes, mirroring the pattern described in eutherian mammals (e.g., mouse, [42]) (S2, S3 and S4 Figs).

### Actively transcribed and elongated telomeres in Dasyuridae germ cells

Remarkably, prominent phosphorylated RNA pol II signals were detected at telomeres in the fat-tailed dunnart (Fig 4A). RNA pol II telomeric signals were predominant in late zygotene and early pachytene (Fig 4A), suggesting that telomeres were actively transcribed during this window. This unique observation has implications for homeostasis of dimorphic dasyurid telomeres. To determine whether short telomeres were elongated in the dunnart during male meiosis, we characterised telomere length by quantitative fluorescent *in situ* hybridisation (Q-FISH) as spermatogenesis progressed. These data were compared with telomere length in tammar and the mouse, as the dynamics of telomere length during mouse male meiosis is known [43].

**Fig 7:**
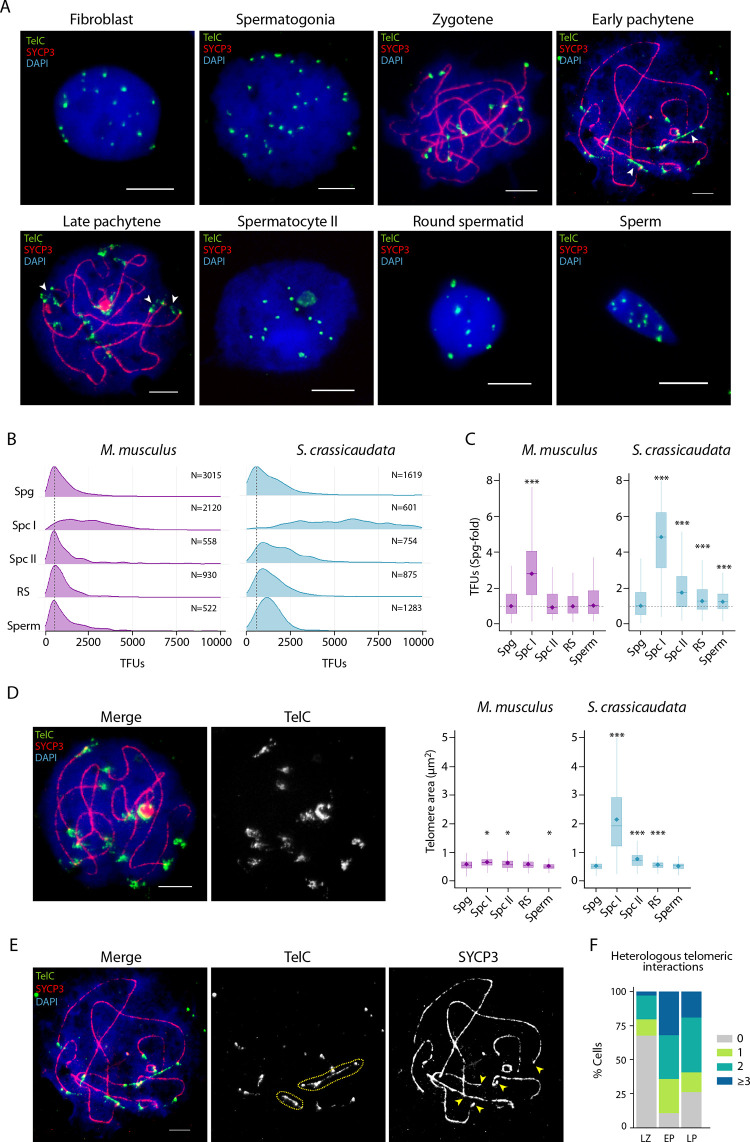
Telomere elongation in the fat-tailed dunnart. (A) Representative Q-FISH images using a TelC probe (green) and an antibody against SYCP3 (red), DNA counter stained with DAPI (blue). White arrows: heterologous telomeric interactions. Scale bar = 10μm. (B) Density plots representing TFUs (Telomere Fluorescence Units) (X-axes) for each cell type in both studied species (mouse and the fat-tailed dunnart). Dashed line: TFU mode for spermatogonia cells. (C) Boxplots representing TFU fold-increase relative to spermatogonia in each species (Wilcoxon pairwise test, ***p<0.001). Dashed line: TFUs median for spermatogonia cells. Boxplots are represented as centre lines (median), box limits (25^th^ and 75^th^ percentiles) and whiskers (largest and lowest data points inside the first and third quartiles plus 1.5 times the interquartile range). Diamonds indicate median value. (D) Left panels: Dunnart late pachytene labelled with an antibody against SYCP3 (red) and a PNA telomere probe (green), DNA counter stained with DAPI (blue). Scale bar = 10μm. Right panels: Boxplots representing telomere area (expressed as μm^2^) in mouse and dunnart germ cells. Wilcoxon pairwise test (*p<0.05, ***p<0.001). Boxplots are represented as in Fig 7C. (E) A representative dunnart pachytene cell labelled with an antibody against SYCP3 (red) and a PNA telomere probe (green), and DNA counter stained with DAPI (blue). Dashed outlines highlight telomeric bridges between heterologous chromosomes. Yellow arrows highlight telomeres involved in heterologous interactions. Scale bar = 10μm. (F) Percentage of cells with different heterologous telomeric interaction events per cell (0, 1, 2 or ≥3) for spermatocytes in late zygotene (N = 34 cells), early pachytene (N = 28 cells) and late pachytene (N = 42 cells) in the fat-tailed dunnart. Cell type legend: LZ: late zygotene, EP: early pachytene, LP: late pachytene.

Fat-tailed dunnart telomeres had a chromatin configuration distinct to those of mouse and tammar wallaby at the equivalent pachytene stages (S5A Fig). Mouse and tammar wallaby showed small discrete telomeric signals, contrasting the larger diffuse fat-tailed dunnart signals that had frequent heterologous telomeric associations (Figs 7, S5 and S6). In addition, tammar wallaby cells showed large heterochromatic interstitial telomeric signals (het-ITSs) (S5B Fig) as previously described [33].

Telomere length (expressed as telomere fluorescence units, TFUs) was measured at all stages of spermatogenesis in the fat-tailed dunnart. This included spermatogonia (N = 1,619 telomeres), spermatocytes I (N = 601 telomeres), spermatocytes II (N = 754 telomeres), round spermatids (N = 875 telomeres) and sperm (N = 1,283 telomeres) (Fig 7A). Cultured fibroblast cells were included as a somatic control. These data were compared with telomere length in the mouse. For mouse, we included spermatogonia (N = 3,015 telomeres), spermatocytes I (N = 2,120 telomeres), spermatocytes II (N = 558 telomeres), round spermatids (N = 930 telomeres) and sperm (N = 522 telomeres) (S5C Fig).

Telomere lengths in dunnart spermatogonia reflect that of somatic cells (cultured fibroblasts), with two populations. In both cell types Q-FISH revealed a bimodal distribution of long and short telomeres, with marked heterogeneity of the long telomere subset (Fig 7B). In primary spermatocytes telomeric signals were larger and more diffuse, losing the bimodal size distribution and reaching their maximum size in late pachytene of approximately a 6-fold increase in TFU (6,044.4 ± 4,500.99) compared to spermatogonia (1,032.03 ± 1,244.15) (Wilcoxon pairwise test, p<0.001) (S5D Fig). This was concomitant with the RNA pol II signals observed on telomeres in primary spermatocytes (Fig 4A). At later stages of spermatogenesis (spermatocytes II, round spermatids and sperm) there were also longer telomeric signals (1,863.05±2,193.17, 1,302.48±1,200.10, 1,262.78±681.68, respectively) than in spermatogonia, indicating that telomeres were elongated throughout prophase I (Wilcoxon pairwise test, p<0.001). In contrast, telomere length in mouse remained stable through spermatogenesis (Fig 7B and 7C), as previously described [43]. Compared to mouse, dunnart had longer telomeres at all stages of spermatogenesis (Fig 7B).

To understand the dynamics of this telomere elongation in dunnart, we expanded our analysis and subdivided primary spermatocytes into late zygotene (N = 82 telomeres), early pachytene (N = 182 telomeres) and late pachytene (N = 337 telomeres). We detected larger telomeric signals in early pachytene (6,867.91±3,810.19) than in zygotene (3,557.41±3,538.72) (Wilcoxon pairwise test, p<0.001) and late pachytene (6,040.33±4,861.80) (Wilcoxon pairwise test, p<0.05) (S5D Fig). Telomeric RNA pol II was most predominant in late zygotene (24% telomeres per cell) and early pachytene (31% telomeres per cell) (Fig 4A), suggesting that although telomere transcription initiates at late zygotene it is not until early pachytene that telomeres reach their maximum size.

To test whether telomere length in dunnart spermatogenesis was associated with open chromatin configurations we measured areas of the telomeric signal (expressed as μm^2^) (Fig 7D). Dunnart telomeric areas were significantly larger in spermatocytes I (2.06±1.82 μm^2^) than in spermatogonia (0.50±0.18 μm^2^), spermatocytes II (0.71±0.31 μm^2^) and sperm (0.59±0.18 μm^2^) (Wilcoxon pairwise test, p<0.001). In contrast, there were not incremental changes in mouse germ cells (Fig 7D). During prophase I in dunnart, telomere area was significantly larger in pachytene (2.30±1.85 μm^2^) than zygotene (0.99±0.64 μm^2^) (Wilcoxon pairwise test, p<0.001), being larger in late pachytene (2.86±1.93 μm^2^) than in early pachytene (1.56± 0.92 μm^2^) (Wilcoxon pairwise test, p<0.001) (S5E Fig).

Finally, we made the surprising observation of chromatin bridges connecting telomeres of heterologous chromosomes (herein heterologous telomere associations) (Figs 7E and S6). Heterologous associations were more prevalent during early pachytene, involving several chromosomes (89% of cells analysed), than zygotene (32% of cells analysed) and late pachytene (74% of cells analysed) (Fig 7F). These heterologous associations were not observed in later stages of meiosis (i.e., spermatocytes II and round spermatids), or in sperm (Fig 7A).

Moreover, we observed terminal regions of homologous chromosomes in which the lateral elements of the synaptonemal complex were not completely synapsed (herein asynapsed telomeres) (S5F Fig). Asynapsed telomeres were visualised from zygotene to late pachytene (between 7–9% of cells analysed) and appeared to encompass the distal region of chromosomes (S5F Fig).

Collectively, our results show that telomere elongation in dunnart spermatocytes takes place during prophase I. This elongation is accompanied by transcription of open telomeric chromatin, and coupled with heterologous telomere associations.

## Discussion

Here we describe key features of meiotic progression in previously uncharacterised marsupial species, with a focus on sex chromosome pairing and silencing strategies, the formation of DSBs and telomeric homeostasis. These results uncover novel mechanisms, deepening our understanding of the regulation of meiotic progression in mammals.

### Differential meiotic sex chromosome pairing and silencing in marsupials

We show how sex chromosome dynamics during marsupial meiosis differs between species, which is most probably associated with sex chromosome structure. Both the fat-tailed dunnart (an Australian dasyurid) and the fat-tailed mouse opossum (an American Didelphidae representative) have a shared conserved X chromosome structure. In contrast, the tammar wallaby (an Australian macropod) presents a larger derived X chromosome that bears a NOR and satellite repeats. We detected that, unlike the fat-tailed dunnart and the fat-tailed mouse opossum X chromosomes (and the Y chromosome in all species), the tammar X displayed signals for active RNA pol II around its chromosomal axis from zygotene to mid pachytene. These results suggest that, unlike in other therian mammals, certain regions in the p- and q-arm of the tammar X chromosome might escape MSCI for much of pachytene. This was despite γH2AX accumulation on both the X and Y chromosomes during the same meiotic stages, implying that γH2AX was not exclusively associated with meiotic gene silencing on the tammar X. Such a pattern signifies at least partial activation of the X chromosome in early/mid pachytene, with implications for marsupial (and mammals more broadly) sex chromosome evolution in context of the ‘persistent Y’ hypothesis [30].

In eutherian mammals (i.e., mice), ectopic expression of Y borne pachytene-lethal (‘executioner’) genes during the MSCI window results in meiotic arrest. If these genes are translocated to an autosome, they escape MSCI and the gamete dies [29,30]. These executioner genes can only be heritably removed from the Y via translocation to the X, where they remain subject MSCI. This poses strong constraint for any Y chromosome with executioner protection to not be lost from the population. Because the tammar X chromosome is partially transcribed through pachytene sub-stages, translocation of executioner genes to the X would not keep them silenced the entirety of pachytene like in other species. There would be nowhere in the genome for them to move away from the Y and remain subject to MSCI. Such translocations would trigger meiotic checkpoints so, essentially, the tammar Y chromosome could be immune from ever being lost, i.e. persistent.

Surprisingly, there was γH2AX signal on both sex chromosomes in early zygotene through mid pachytene, when the X chromosome in tammar was also enriched for active RNA pol II. This suggests that γH2AX has an additional function distinct from its canonical role of meiotic gene silencing, potentially related to the tight paring of sex chromosomes in the absence of a PAR. It is well known that in eutherians the formation of DSBs (catalysed by SPO11) induces a first wave of γH2AX by the ATM/ATR pathway in early prophase I [21,24,44]. γH2AX helps recruit proteins involved in the DNA damage response mechanism [45,46], including: RPA [37], DMC1 [40] and RAD51 [39], among others. In response to unsynapsed chromatin regions, a second wave of γH2AX coats both X and Y chromosomal axes, resulting in MSCI [20,47]. This second wave is ATR-dependent but independent of SPO11 [45,46], thus not related to the formation of DSBs.

In the marsupial species studied here, the first wave of γH2AX observed in early and late zygotene was articulated as a faint signal in the whole nucleus. In the second wave, however, the γH2AX signal was stronger and restricted to the sex chromosomes during late prophase I, corresponding with the formation of the DP and the initiation of MSCI. In the absence of a PAR in marsupials, perhaps this second wave of γH2AX plays a structural role in maintaining sex chromosome association, in addition to the DP. That is, γH2AX accumulates on the X and Y early before pairing, is maintained on asynapsed sex chromosomes in close proximity during late pachytene, and then all through the first meiotic division, perhaps contributing to their proper segregation. This view is in agreement with previous observations of γH2AX-positive chromatin filaments in sex chromosomes at metaphase I in eutherian species with neo- or asynaptic sex chromosomes [48–50], suggesting that epigenetic MSCI features might play a role in the segregation of achiasmate sex chromosomes.

### Low levels of γH2AX and the formation of DSBs

The faint γH2AX signal observed in the three marsupial species during the first wave of γH2AX in early prophase I, suggests an association with low recombination rates. Although variable between species, between 200–300 DSBs per cell (mean values) occur genome-wide during leptotene in eutherian mammals [12,51–53]. Despite differences in replacement dynamics (probably due to intrinsic intra-specific variability), we detected far fewer (less than 150 per cell) RPA and RAD51 foci (a proxy for DSBs) in marsupials than in eutherians. This supports previous genetic linkage maps and modelling analysis that reported low recombination rates in marsupials [3,41,54].

We propose that the low levels of recombination rates observed in marsupials result from a reduction of DSB formation, and consequently a genome wide reduction of γH2AX in early prophase I. Eutherian mammals have diploid numbers ranging from 2n = 6 to 2n = 102 [5], whereas marsupials are characterised by low diploid numbers (ranging from 2n = 10 to 2n = 32, [35]) that could influence the low recombination rates observed. It is known that the final number of crossovers can be modulated by factors such as chromosome axis length and DNA chromatin loop length [12,55–57].

These two events (DNA loop size and the initial number of DSBs formed in early stages of meiosis) are in fact interconnected (i.e., [56]). In bovids, for example, the number of crossovers and DSBs per cell correlates positively with synaptonemal complex length and chromatin loop size [12]. That is, species with a low number of chromosomes display shorter chromosomal axes, longer DNA loops and a low number of DSBs, and hence crossovers. Therefore, it can be expected that marsupial species with low diploid numbers (such as those included in this study) would also have lower numbers of DSBs in leptotene than do eutherian mammals with higher diploid numbers, as reduced crossovers number will be sufficient to ensure the formation of the obligatory chiasmata.

### Meiotic telomeric elongation in fat-tailed dunnart males

Remarkably, we also detected that telomeric length was reprogrammed during male meiosis in the fat-tailed dunnart, a unique telomeric elongation strategy previously undescribed for mammals. No such phenomenon has been previously observed in human and mouse oocytes or spermatocytes, nor any other mammal [43,58–62]. In male dasyurids, the Y chromosome is characterised by long telomeres, whereas X chromosomes have short telomeres, and all autosome pairs have dimorphic telomere length. This pattern is indicative of paternally inherited long telomeres [33], begging for a mechanism to explain such pattern.

**Fig 8:**
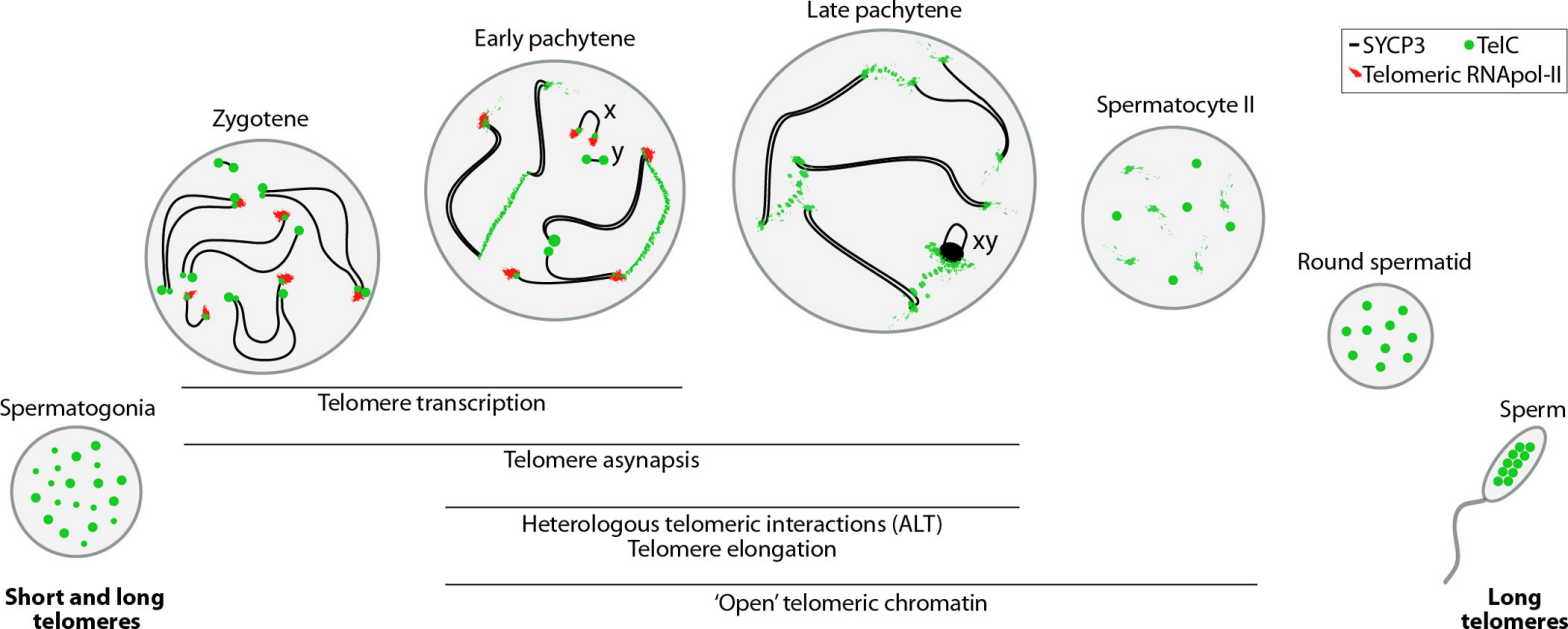
Proposed model for telomere elongation in the fat-tailed dunnart. Spermatogonia present two populations of telomeres, short and long. Telomere RNA pol II appears at zygotene, being predominant in early pachytene. Telomere transcription resulted in telomere elongation, which was accompanied by heterologous telomeric interactions and open chromatin states through late pachytene. Telomere elongation resulted in a homogenous population of long telomeres in round spermatids and sperm. Legend: TelC–telomeric probe, ALT—Alternative Lengthening of Telomeres.

Here we demonstrate that paternal control of telomere length indeed takes place during prophase I of meiosis. Moreover, telomere elongation was accompanied by: (i) the transcription of telomeres, (ii) telomeric associations between heterologous chromosomes, (iii) ‘open’ chromatin configurations at telomeres and (iv) asynapsed telomeres. Here we propose a meiotic telomeric elongation model (Fig 8) that reconciles telomerase activity, telomere transcription and alternative lengthening of telomeres (ALT) [63,64] (exemplified by telomeric associations) to explain dasyurid telomere homeostasis.

During meiotic prophase I in eutherian oocytes and spermatocytes, telomeres are actively transcribed from sub-telomeric regions producing long non-coding telomeric repeat-containing RNA (TERRA) [65,66]. This expression initiates in spermatogonia, increases with spermatogenesis progression, reaches a maximum in spermatocytes II, and finally decreases at the beginning of spermiogenesis (i.e., round spermatids) [43,61]. In fission yeast (which have human-like telomeres) shortened telomeres have high TERRA expression, stimulating telomerase recruitment and activity [67]. Telomerase is active in the testes of Tasmanian devil (another dasyurid representative with heteromorphic telomeres, [33]), suggesting that it is active during male meiosis and extending short telomeres. Our observation of active RNA pol II accumulation at the telomeres in dunnart spermatocytes is compatible with TERRA expression, and subsequent telomerase recruitment.

We also observed open chromatin configurations at chromosome ends in dunnart spermatocytes, which fits with a model proposed by Moravec and collaborators [67]. Moravec et al. proposed that telomerase can discriminate between telomeres of different lengths based on chromatin state. Long telomeres are maintained in a closed chromatin state producing limited amounts of TERRA, whereas short telomeres present an open chromatin state, permitting telomerase accessibility and telomere elongation.

The open chromatin states of dunnart telomeres were coupled with heterologous telomeric associations in early pachytene, which supports a putative role for ALT, as previously speculated [34]. ALT is a homologous recombination-based mechanism, initially described in cancer cells but also detected in pluripotent stem cells, to maintain long telomeres that are heterogeneous in size [68,69]. In ALT telomeres, RPA/RAD51/HOP2-MND1-mediated homology searches can induce inter-telomere recombination [70]. Moreover, RNA polymerase activity is required for ALT [71], which can occur simultaneously with telomerase activity [72]. This, together with the fact that chromatin compaction at ALT telomeres is reduced [73] and accompanied by higher levels of TERRA [74], suggest that telomere homeostasis in dunnart is a complex phenomenon involving different mechanisms.

But what is the physiological relevance of having this unique strategy for telomere maintenance in marsupials? It was initially suggested that dasyurid males having long telomeres could be related to the stress that results from semelparity [34], a short intense breeding season that can lead to male mortality, which has evolved independently in several dasyurid species [75]. As accelerated telomeric shortening can be associated to life stress [76], and the short breeding season of semelparous species certainly represents a period of extreme stress for males, telomere elongation during spermatogenesis could compensate for this critical loss. But as dunnart is not semelparous, this explanation seems unlikely. Alternatively, ALT might predispose dasyurids to evolving the semelparous reproductive strategy observed in some species [34]. Additionally, dasyurids have a proposed genetic predisposition to lymphomas [77], so further exploration of ALT in the germline could provide new insight into the role that telomere homeostasis plays in the high incidence of tumours reported for species within Dasyuridae (i.e., devil facial tumour).

Overall, the proposed model for telomere elongation in the fat-tailed dunnart (Fig 8) explains how short telomeres can be reliably transformed into long telomeres within one meiotic cell division in the male germ line. More importantly, our evidence for alternative lengthening of telomeres in a mammalian germline opens new avenues to explore telomere homeostasis mechanisms (distinct from telomerase) in a non-cancer setting.

## Materials & methods

### Ethics statement

All animals were held and tissues collected under appropriate permits, and experiments approved by each Universities Animal Experimentation Ethics Committees (University of Melbourne, Universidad de Chile, Universidad Autónoma de Madrid and Universitat Autònoma de Barcelona) in accordance with animal ethics guidelines.

### Animals and cell line

Tammar wallaby males (N = 2, *Macropus eugenii*) were collected from wild populations originating on Kangaroo Island (South Australia) that were held in a breeding colony in Melbourne (Victoria, Australia). Fat-tailed dunnart males (N = 2, *Sminthopsis crassicaudata)* were collected from breeding colonies in Melbourne (Victoria, Australia). Fat-tailed mouse opossum males (N = 3, *Thylamys elegans*) were collected from wild populations in the central region of Chile. Mouse testes samples were obtained from adult C57BL/6 male mice (N = 2, 90–120 days old).

A primary fibroblast cell line was established from one of the fat-tailed dunnart males following standard procedures. Briefly, a sample of connective tissue from ribs was washed in 1xPBS supplemented with an antibiotic-antimycotic solution (100U/ml penicillin, 100μg/ml streptomycin, 50μg/ml gentamicin and 0.25μg/ml amphotericin B). Cultures were established by disaggregating tissue with a scalpel blade and resuspending cells in AmnioMAX. Cell cultures were incubated at 35°C in 5% CO_2_.

### Spermatocyte spreads and immunofluorescence

Testicular biopsies were obtained immediately after dissection and processed as previously described [9,78] in order to obtain spermatocyte spreads. Briefly, a piece of the testicular biopsy was gently cut up on a slide in 1xPBS. Subsequently, 1% Lipsol was added and incubated for 30 minutes at room temperature. Then, a fixative solution containing 4% paraformaldehyde was added, and slides were kept in a humid chamber. After two hours, slides were washed in 1% Photo-Flo solution and further processed for immunofluorescence, or frozen at -20°C until use. Additionally, samples were processed for squashing as previously described [79].

Immunostaining of meiocytes was performed as previously described [3] using the following primary antibodies: rabbit antibody against SYCP3 (#ab15093, Abcam, 1:200 dilution), goat antibody against SYCP3 (#SC-33874, Santa Cruz Biotechnology, 1:50), human calcinosis, Raynaud’s phenomenon, oesophageal dysfunction, sclerodactyly and telangiectasia (CREST) serum (a kind gift of M. Fritzler, 1:50 dilution), rabbit antibody against SYCP1 (#ab15087, Abcam, 1:100 dilution), mouse antibody against RNA pol II phosphorylated at serine 5 (#5408, Abcam, 1:400 dilution), mouse antibody against RNA pol II phosphorylated at serine 2 (#24758, Abcam, 1:100 dilution), rabbit antibody against γH2AX (#H5912, Sigma-Aldrich, 1:100 dilution), mouse antibody against γH2AX (#05–636,Upstate, 1:1000 dilution), rabbit antibody against RAD51 (#PC130, Calbiochem, 1:50 dilution), rabbit antibody against RPA (#10359, Abcam, 1:50 dilution). Fluorochrome-conjugated secondary antibodies were used for detection (all from Jackson ImmunoResearch Laboratories, 1:200). Antibodies were diluted in PBST (0.05% Tween in PBS). Primary antibodies were incubated overnight at 4°C or at room temperature in a humid chamber. Secondary antibodies were incubated for 1 h at 37°C in a humid chamber. After washing away excess secondary antibodies, DNA was counterstained with anti-fade solution (Vectashield) containing 8μg/ml DAPI (4’,6’-diamidino-2-phenylindole).

### Quantitative-Fluorescence *in situ* Hybridisation

After immunofluorescence, spermatocyte spreads were subjected to quantitative-fluorescence *in situ* hybridisation (Q-FISH) analysis using a peptide nucleic acid (PNA) probe complementary to telomere G-rich strand as previously described [43]. Briefly, cells were air-dried, fixed in 4% formaldehyde, and their cytoplasm removed with 0.005% pepsin. After ethanol dehydration, cells were hybridized with TelC PNA probe FAM-conjugated (#F1001, Panagene), complementary to G-rich strand, according to the manufacture instructions. Finally, cells were washed using two different solutions carrying 1xPBS and 2xSSC mixed with 0.1% Tween-20 and stained with 8μg/ml DAPI diluted in Vectashield. For each experiment, an internal control consisting of mouse germ cells with known telomere length [43,80] was included. In the fat-tailed dunnart, an additional step of nuclear decondensation of spermatozoa [81] was included before Q-FISH. Briefly, slides were washed in 2xSSC for 3 minutes twice and dehydrated by an increasing ethanol battery (EtOH at 70%, 90% and 100%). After letting the slides dry, samples were submerged in dithiothreitol (DTT) solution (5mM DTT, 1% Triton X-100, 50mM Tris) for 8 minutes at 37°C. Finally, samples were washed with 2xSSC twice, after which slides were ready to perform Q-FISH.

### Microscopy and image analysis

IF/Q-FISH treated slides were analysed using an epifluorescence microscope (Axiophot, Zeiss) equipped with a camera (ProgRes CS10plus, Jenoptik) and suitable emission filters (DAPI, FITC and Cy3). Images were captured with 63x lens and using ACO XY program (A. Coloma, Open Microscopy). Variables including exposure and gain were constant between experiments depending on the selected channel (gain was maintained at 6.46 dB in all channels), whereas selected exposures were 450ms for green (495 nm excitation wavelength), 600–800ms for red (548 nm excitation wavelength), 40–80ms for blue. For squash preparations, additional observations were made on an Olympus BX61 microscope equipped with a motorized Z axis. Images were captured with an Olympus DP72 digital camera using the Cell-F software (Olympus, Hamburg, Germany). Squashed spermatocytes were photographed at 0.2μm intervals. The resulting stack images were processed using the public domain software ImageJ (National Institutes of Health, USA; http://rsb.info.nih.gov/ij) and Adobe Photoshop 7.0.

Transcription along X chromosome in the tammar wallaby was analysed using the ImageJ software. For each X chromosome, RNA pol II signals on the synaptonemal complex (i.e., SYCP3 signal) were recorded as a relative position from the centromere, expressed as percentage of the synaptonemal complex length of each arm. Only completely stretched X chromosomes in late zygotene and early pachytene cells were included in the analysis.

### Telomere length quantification

Telomere intensities were obtained by measuring spot optical densities using the TFL-TeloV2-2 software [82]. Only cells with a well-defined shape and background were selected for analysis. Briefly, all green channel captures (TelC signal) were converted to 8-bit format for processing in ImageJ software. Telomere intensities were obtained by measuring spot optical densities as arbitrary telomere fluorescent units (TFU). The results were evaluated, corrected, and classified in different cell types following previous studies [43]. Additionally, spot area outputs (provided in pixels) were converted to μm and used for further statistical analyses.

### Statistical analysis

Statistical methods and p-values are displayed in each plot or listed in the figure legends. In brief, data is represented as mean ± standard deviation (SD). All box-and-whisker plots are represented as centre lines (median), box limits (interquartile range; 25^th^ and 75^th^ percentiles) and whiskers (largest and lowest data points inside the first and third quartiles plus 1.5 times the interquartile range).

Statistical significances for the DSB analysis as RPA and RAD51 foci were determined using two-sided Mann-Whitney U-tests since data was not normally distributed (Shapiro Wilk test, p>0.05), first applying the Fisher’s F test of homogeneity of variances. Moreover, the Wilcoxon’s sum rank test was used to compare telomere fluorescent units between cell types and the Pearson’s *χ*^2^ test to compare X chromosome transcription per chromosome conformation. For all tests, statistical significance was considered for p<0.05.

## Supporting information

S1 Fig Pairing dynamics during prophase I. (A) Leptotene spermatocytes spreads labelled with an antibody against SYCP3 (green) and labelling the DNA with DAPI (blue) for (i) the tammar wallaby, (ii) the fat-tailed dunnart and (iii) the fat-tailed mouse opossum. (B-D) Examples of spread spermatocytes in prophase-I labelled with antibodies against SYCP3 (green) and SYCP1 (red) for (B) the tammar wallaby, (C) the fat-tailed dunnart and (D) the fat-tailed mouse opossum. Scale bar = 10μm.
(TIF)

S2 Fig RPA and RAD51 dynamics during prophase I in the tammar wallaby. Tammar spread spermatocytes in prophase-I labelled with antibodies against SYCP3 (green), RAD51 (red) and RPA (red). DNA counter stained with DAPI (blue). The positions of identifiable sex chromosomes are encircled in yellow. Scale bar = 10μm.
(TIF)

S3 Fig RPA and RAD51 dynamics during prophase I in the fat-tailed dunnart. Fat-tailed dunnart spread spermatocytes in prophase-I labelled with antibodies against SYCP3 (green), RAD51 (red) and RPA (red). DNA counter stained with DAPI (blue). The positions of identifiable sex chromosomes are encircled in yellow. Scale bar = 10μm.
(TIF)

S4 Fig RPA and RAD51 dynamics during prophase I in the fat-tailed mouse opossum. Fat-tailed mouse opossum spread and squash spermatocytes in prophase-I labelled with antibodies against SYCP3 (green), RPA (red) and RAD51 (red). The positions of identifiable sex chromosomes are encircled in yellow. Scale bar = 10μm.
(TIF)

S5 Fig Telomeric homeostasis in Australian marsupials during spermatogenesis. (A) Mouse, tammar wallaby and fat-tailed dunnart spread pachytene cells labelled with an antibody against SYCP3 (red) and a PNA telomere probe (green), DNA counter stained with DAPI (blue). Dashed outlines: large heterochromatic interstitial telomeric signals (het-ITSs). (B) Tammar metaphase chromosomes labelled with a PNA telomere probe (green) showing het-ITSs in all chromosomes as previously described [33,83]. DNA counter stained with DAPI (blue). (C) Representative Q-FISH images using a TelC probe (green) and antibody against SYCP3 (red) for different cell types of mouse spermatogenesis. DNA counter stained with DAPI (blue). Scale bar = 10μm. (D) Density and box plots representing telomere length as TFUs in dunnart primary spermatocytes. Boxplots are represented as in Fig 7C. (E) Boxplots representing telomere area (expressed as μm^2^) in dunnart primary spermatocytes. Boxplots are represented as in Fig 7C. Wilcoxon pairwise test (*p<0.05, ***p<0.001). Cell type legend: Z, zygotene; EP, early pachytene; LP, late pachytene. (F) Left panels: Images of dunnart pachytene cells labelled with an antibody against SYCP3 (green), DNA counter stained with DAPI (blue). Scale bar = 10μm. Insets represent asynapsed telomeres (yellow arrows). Right panels: Percentage of cells with asynapsed telomeres for spermatocytes in late zygotene (N = 58 cells), early pachytene (N = 70 cells) and late pachytene (N = 66 cells) in the fat-tailed dunnart. Cell type legend: LZ: late zygotene, EP: early pachytene, LP: late pachytene.
(TIF)

S6 Fig Heterologous telomeric interactions in the fat-tailed dunnart. Examples of spread pachytene cells in dunnart labelled with an antibody against SYCP3 (red) and a PNA telomere probe (green), DNA counter stained with DAPI (blue). Dashed outlines: telomeric bridges between heterologous chromosomes. Yellow arrows: telomeres involved in heterologous interactions. Scale bar = 10μm.
(TIF)

S1 Table Transcription dynamics during prophase I in the tammar wallaby. Number of cells with different transcriptional patterns and different X chromosome conformations in zygotene (N = 41 cells), early pachytene (N = 19 cells), mid pachytene (N = 49 cells) and late pachytene (N = 81 cells)
(XLSX)

S1 Data Table containing the raw data underlying boxplots and graphs displayed in main and supporting figures. (XLSX)
