# Supplementary figures and images for: Strategies for meiotic sex chromosome dynamics and telomeric elongation in Marsupials
Source: PLoS Genet. 2022 Feb 7;18(2):e1010040. doi: 10.1371/journal.pgen.1010040 (PMC8853506; doi:10.1371/journal.pgen.1010040)

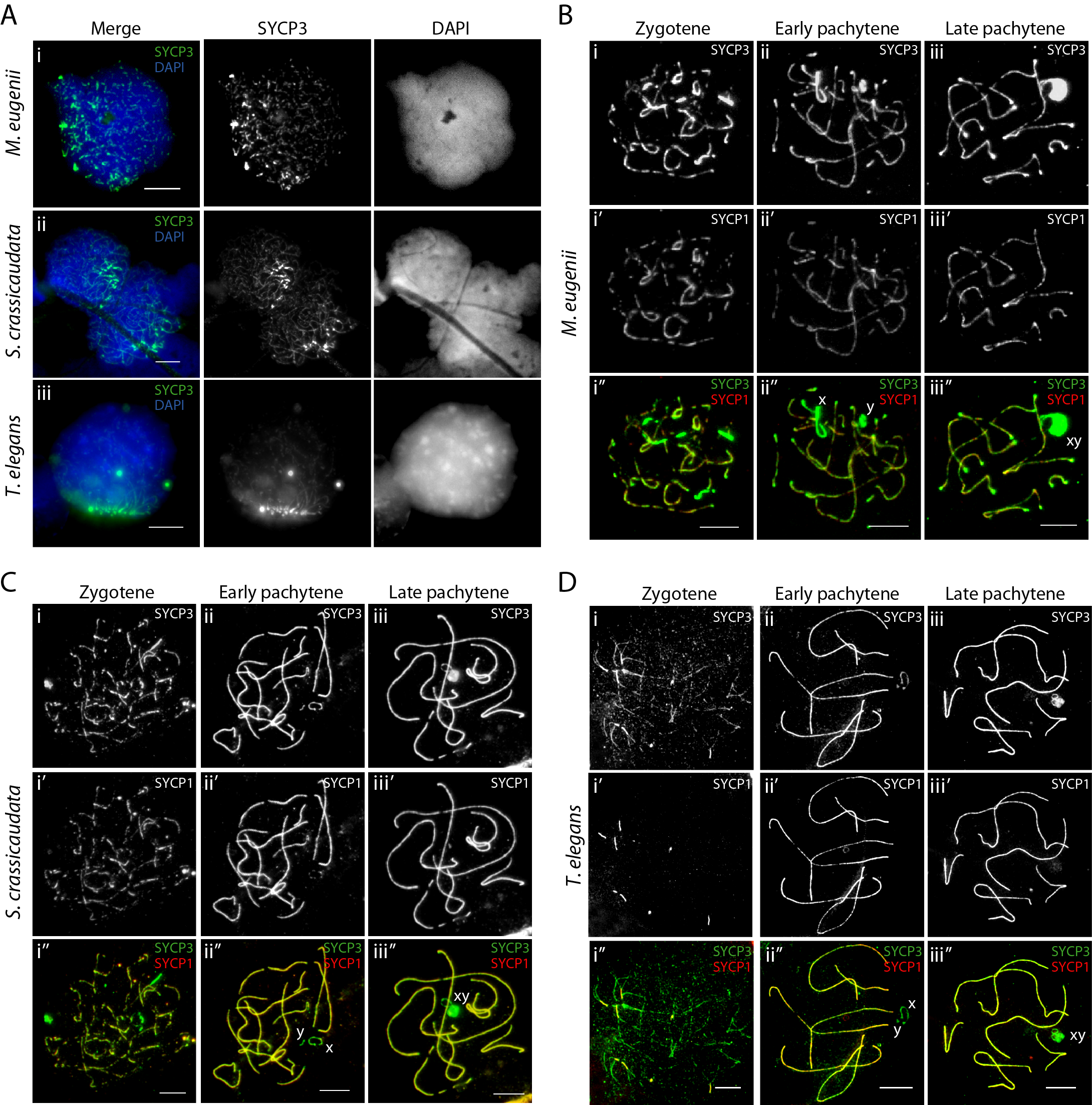

Supplement: S1 Fig — (A) Leptotene spermatocytes spreads labelled with an antibody against SYCP3 (green) and labelling the DNA with DAPI (blue) for (i) the tammar wallaby, (ii) the fat-tailed dunnart and (iii) the fat-tailed mouse opossum. (B-D) Examples of spread spermatocytes in prophase-I labelled with antibodies against SYCP3 (green) and SYCP1 (red) for (B) the tammar wallaby, (C) the fat-tailed dunnart and (D) the fat-tailed mouse opossum. Scale bar = 10μm. (TIF) [file pgen.1010040.s001.tif]

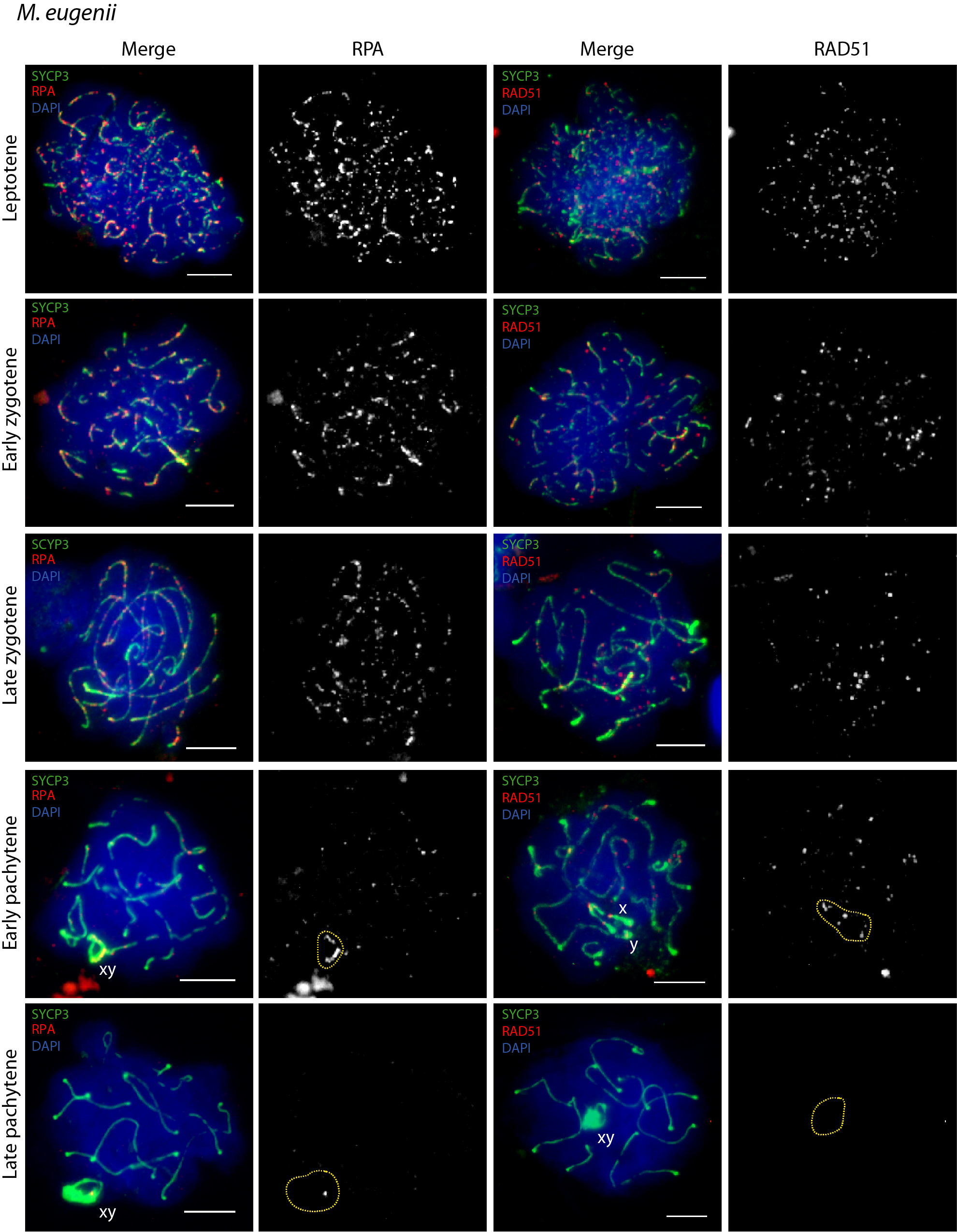

Supplement: S2 Fig — Tammar spread spermatocytes in prophase-I labelled with antibodies against SYCP3 (green), RAD51 (red) and RPA (red). DNA counter stained with DAPI (blue). The positions of identifiable sex chromosomes are encircled in yellow. Scale bar = 10μm. (TIF) [file pgen.1010040.s002.tif]

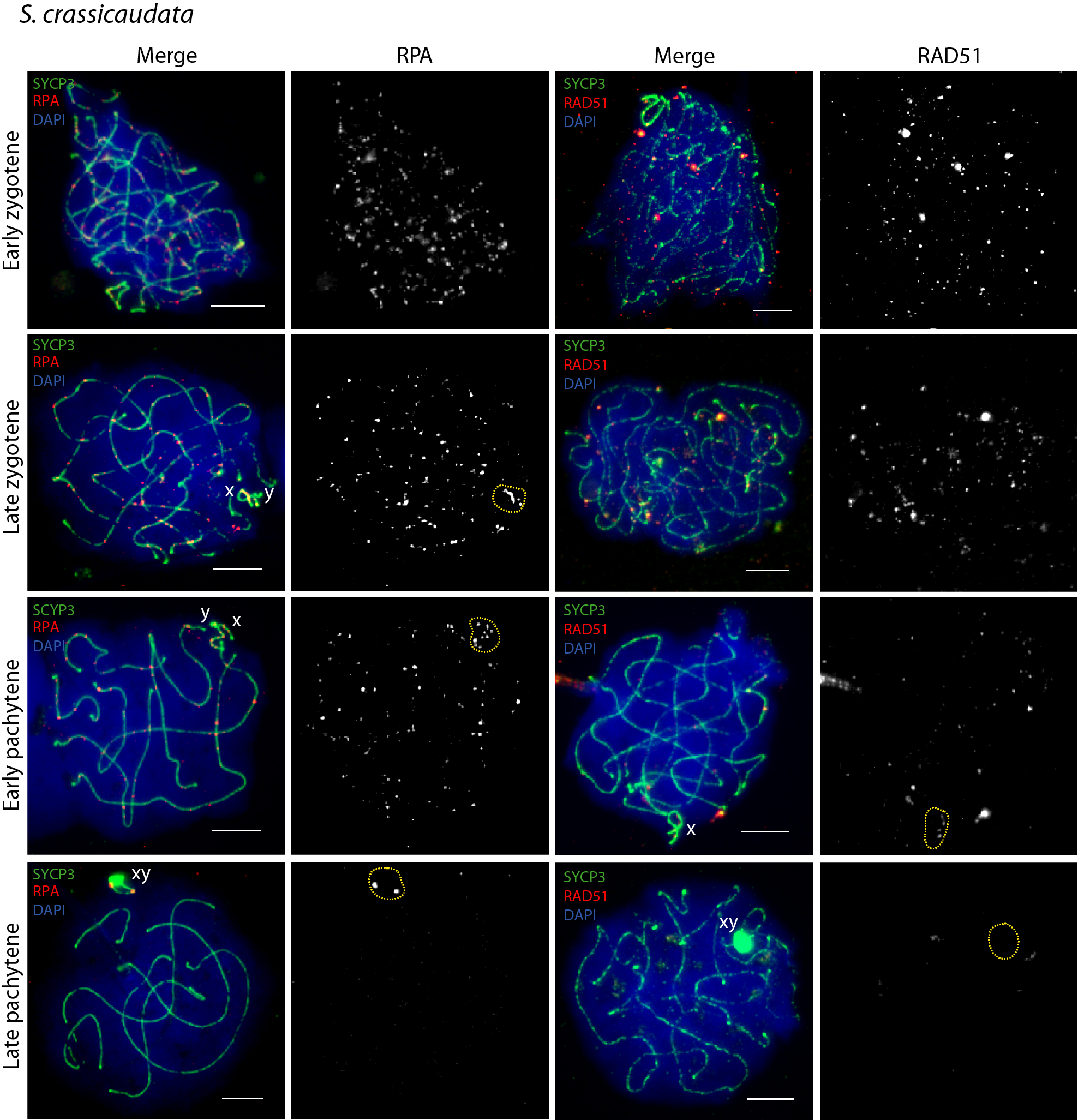

Supplement: S3 Fig — Fat-tailed dunnart spread spermatocytes in prophase-I labelled with antibodies against SYCP3 (green), RAD51 (red) and RPA (red). DNA counter stained with DAPI (blue). The positions of identifiable sex chromosomes are encircled in yellow. Scale bar = 10μm. (TIF) [file pgen.1010040.s003.tif]

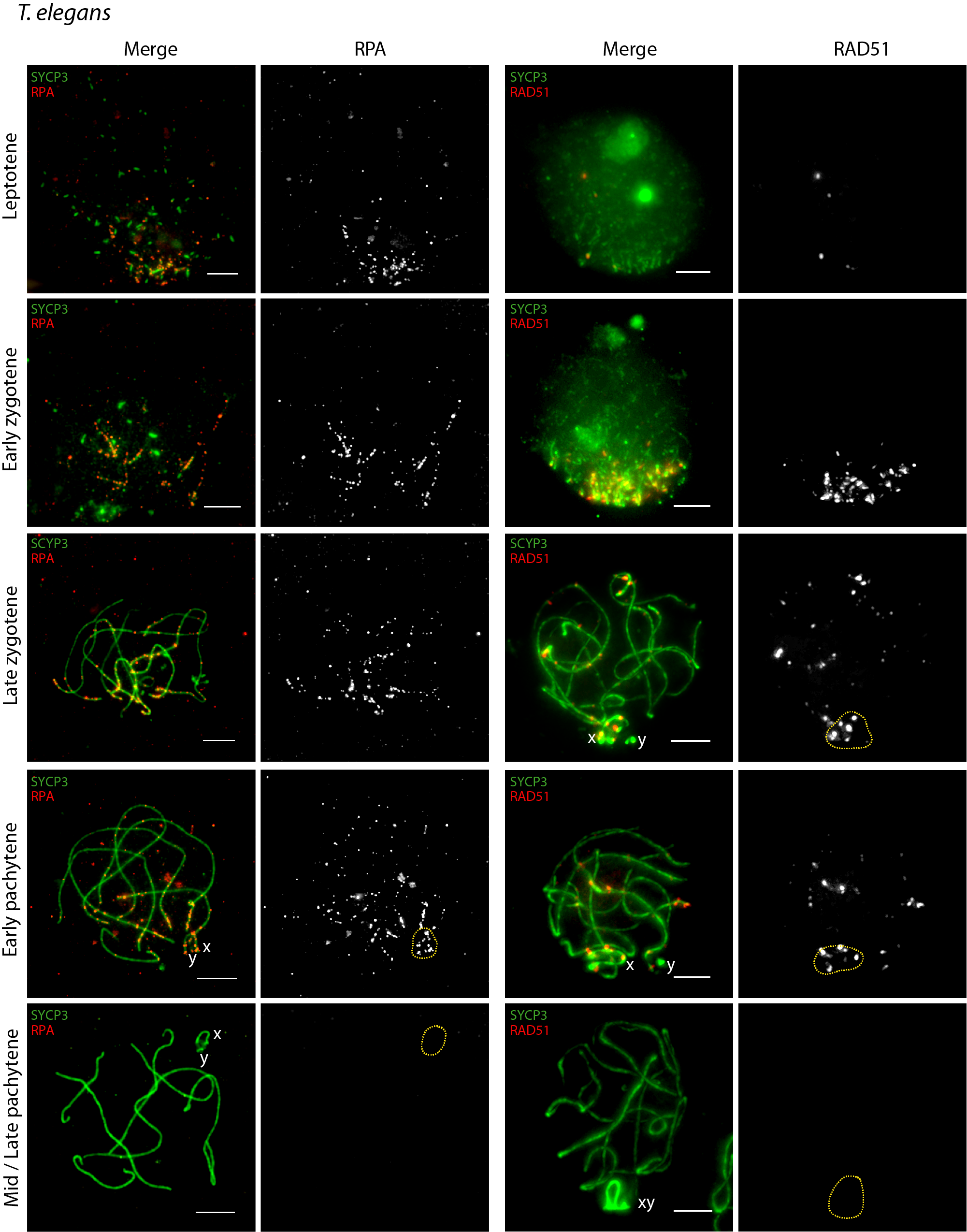

Supplement: S4 Fig — Fat-tailed mouse opossum spread and squash spermatocytes in prophase-I labelled with antibodies against SYCP3 (green), RPA (red) and RAD51 (red). The positions of identifiable sex chromosomes are encircled in yellow. Scale bar = 10μm. (TIF) [file pgen.1010040.s004.tif]

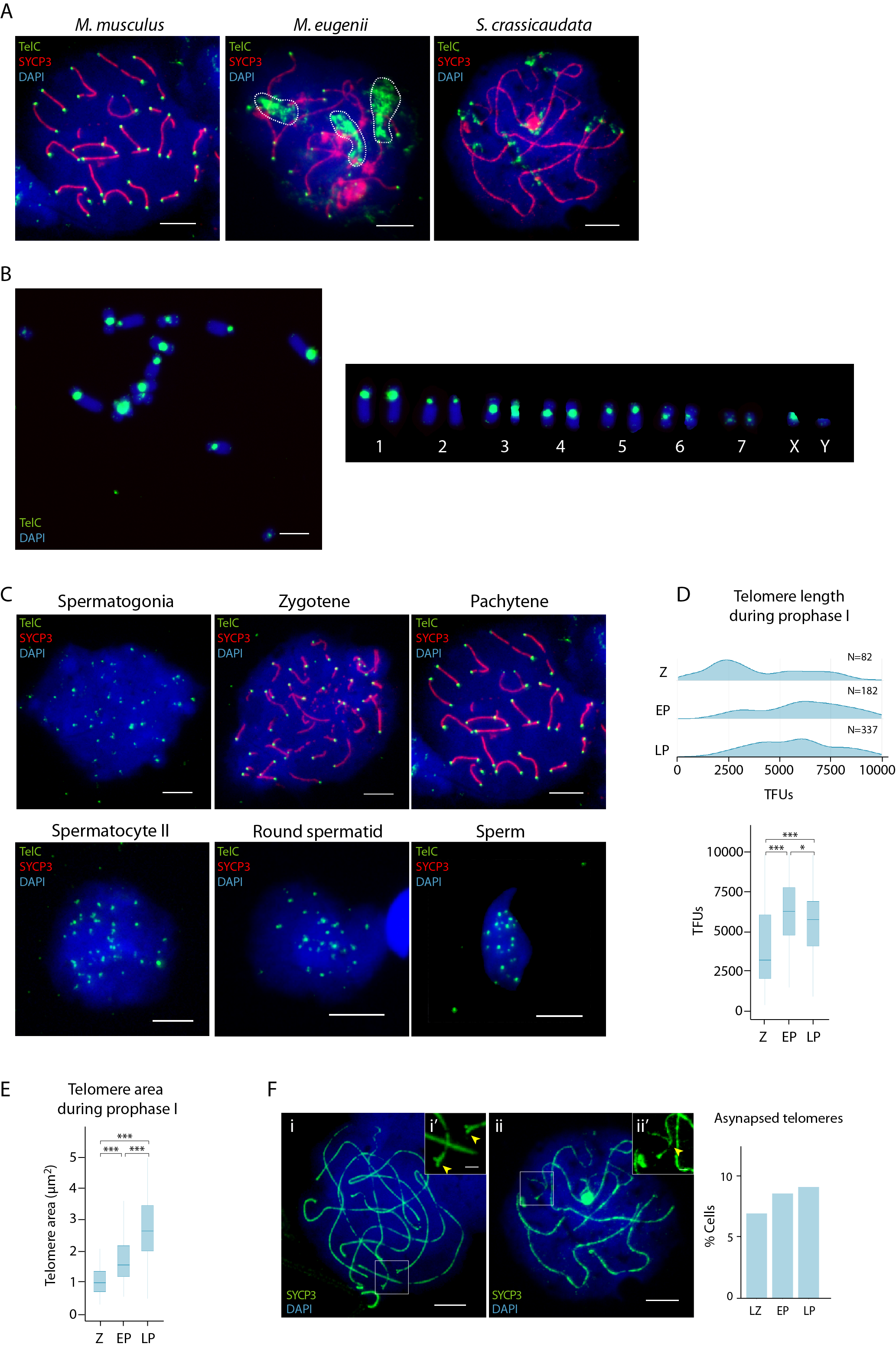

Supplement: S5 Fig — (A) Mouse, tammar wallaby and fat-tailed dunnart spread pachytene cells labelled with an antibody against SYCP3 (red) and a PNA telomere probe (green), DNA counter stained with DAPI (blue). Dashed outlines: large heterochromatic interstitial telomeric signals (het-ITSs). (B) Tammar metaphase chromosomes labelled with a PNA telomere probe (green) showing het-ITSs in all chromosomes as previously described [33,83]. DNA counter stained with DAPI (blue). (C) Representative Q-FISH images using a TelC probe (green) and antibody against SYCP3 (red) for different cell types of mouse spermatogenesis. DNA counter stained with DAPI (blue). Scale bar = 10μm. (D) Density and box plots representing telomere length as TFUs in dunnart primary spermatocytes. Boxplots are represented as in Fig 7C. (E) Boxplots representing telomere area (expressed as μm2) in dunnart primary spermatocytes. Boxplots are represented as in Fig 7C. Wilcoxon pairwise test (*p<0.05, ***p<0.001). Cell type legend: Z, zygotene; EP, early pachytene; LP, late pachytene. (F) Left panels: Images of dunnart pachytene cells labelled with an antibody against SYCP3 (green), DNA counter stained with DAPI (blue). Scale bar = 10μm. Insets represent asynapsed telomeres (yellow arrows). Right panels: Percentage of cells with asynapsed telomeres for spermatocytes in late zygotene (N = 58 cells), early pachytene (N = 70 cells) and late pachytene (N = 66 cells) in the fat-tailed dunnart. Cell type legend: LZ: late zygotene, EP: early pachytene, LP: late pachytene. (TIF) [file pgen.1010040.s005.tif]

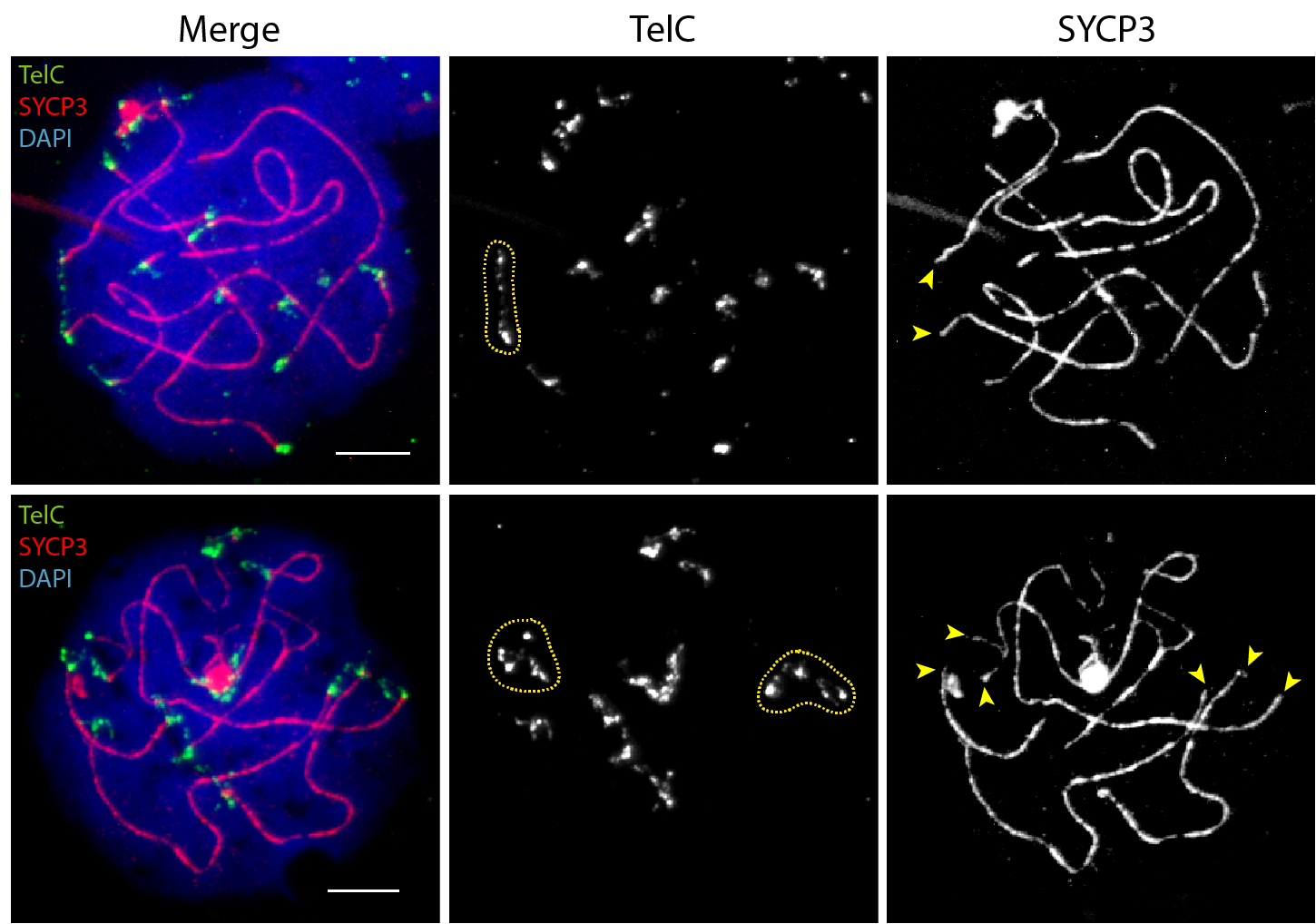

Supplement: S6 Fig — Examples of spread pachytene cells in dunnart labelled with an antibody against SYCP3 (red) and a PNA telomere probe (green), DNA counter stained with DAPI (blue). Dashed outlines: telomeric bridges between heterologous chromosomes. Yellow arrows: telomeres involved in heterologous interactions. Scale bar = 10μm. (TIF) [file pgen.1010040.s006.tif]
